# Advancing non-small cell lung cancer treatment: the power of combination immunotherapies

**DOI:** 10.3389/fimmu.2024.1349502

**Published:** 2024-07-02

**Authors:** Yuanlin Wu, Guangmao Yu, Ketao Jin, Jun Qian

**Affiliations:** ^1^ Department of Thoracic Surgery, Shaoxing People’s Hospital, Shaoxing, Zhejiang, China; ^2^ Department of Gastrointestinal, Colorectal and Anal Surgery, Affiliated Hangzhou First People's Hospital, School of Medicine, Westlake University, Hangzhou, Zhejiang, China; ^3^ Department of Colorectal Surgery, Xinchang People’s Hospital, Affiliated Xinchang Hospital, Wenzhou Medical University, Xinchang, Zhejiang, China

**Keywords:** non-small cell lung cancer, immunotherapy, combination therapy, cancer vaccines, tumor microenvironment

## Abstract

Non-small cell lung cancer (NSCLC) remains an unsolved challenge in oncology, signifying a substantial global health burden. While considerable progress has been made in recent years through the emergence of immunotherapy modalities, such as immune checkpoint inhibitors (ICIs), monotherapies often yield limited clinical outcomes. The rationale behind combining various immunotherapeutic or other anticancer agents, the mechanistic underpinnings, and the clinical evidence supporting their utilization is crucial in NSCLC therapy. Regarding the synergistic potential of combination immunotherapies, this study aims to provide insights to help the landscape of NSCLC treatment and improve clinical outcomes. In addition, this review article discusses the challenges and considerations of combination regimens, including toxicity management and patient selection.

## 1 Introduction

**Figure 1:**
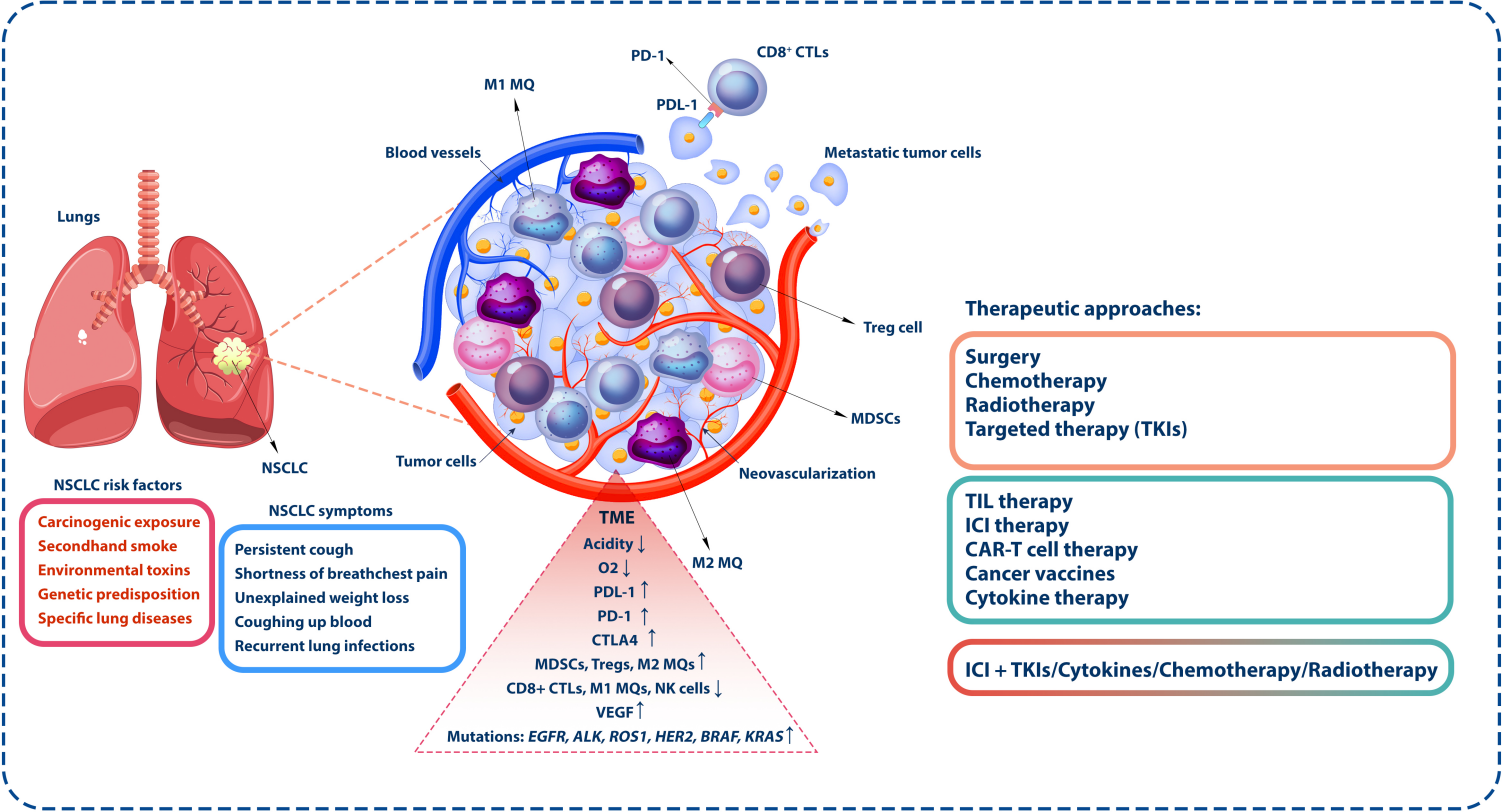
Tumor microenvironment in NSCLC. The inhibitory TME encompasses diverse immune system cells, including inhibitory cells and those with antitumor activity. MDSCs, Tregs, and M2 macrophages are notable among the inhibitory and tumor-associated cells. In contrast, antitumor cells such as NK and CD8^+^ cytotoxic T lymphocytes (CTLs) are specifically responsible for targeting and eliminating tumor cells. Conditions such as reduced oxygen concentration and heightened acidity prevail within the TME. Furthermore, tumor cells express inhibitory molecules like PD-1, which interact with PD-L1 on the surface of CTLs, diminishing the antitumor activity of these cells, which is the base of ICI therapy. However, it is noteworthy that monotherapy and conventional therapies such as surgery, chemotherapy, targeted therapies, radiotherapy, or immunotherapeutic methods exhibit limited effectiveness. Based on the available evidence, a more favorable strategy is combination therapy. This approach seeks to synergize various treatment modalities, enhancing their collective impact and providing a more comprehensive and effective response to the complexities of the TME. NSCLC, non-small cell lung cancer; TME, tumor microenvironment; MDSCs, myeloid-derived suppressor cells; Tregs, regulatory T cells; NK, natural killer; PD-1, programmed cell death protein-1; PD-L1, programmed death-ligand 1; ICI, immune checkpoint inhibitor.

Non-small cell lung cancer (NSCLC) is a prominent reason for cancer-related mortality worldwide, noticeably impacting public health (1). While conventional therapies like surgery, chemotherapy, and radiation therapy have successfully managed NSCLC, recent years have witnessed a paradigm switch to therapeutic strategies (2). Immunotherapeutic methods, particularly immune checkpoint inhibitors (ICIs), have emerged as a potent anticancer approach for NSCLC treatment, recommending hypothetical durable responses in a subset of patients (3). Despite the brightness surrounding immunotherapies, clinical evidence has revealed that monotherapies often yield imperfect outcomes, with only a fraction of patients achieving long-term advantages (4). The complexity of the tumor microenvironment (TME), immune evasion mechanisms, and the heterogeneity of NSCLC are among the most significant challenges associated with inefficient antitumor immune responses observed following mono-immunotherapies (5). Several tumor-supportive immune cells, such as regulatory T cells (Tregs), M2 macrophages, and myeloid-derived suppressor cells (MDSCs), could suppress antitumor immune responses. Hypoxic conditions and high acidity are other features of the tumor milieu (6) (**Figure 1**).

Consequently, combination immunotherapies can be profitable ways to overcome these challenges and limitations. This approach proposes concurrently employing the synergistic potential of various immunotherapeutic agents, affecting multiple aspects of the immune response and TME (7). Combining immunotherapeutic agents with other anticancer approaches aims to boost the patient’s immune system, enhancing its ability to recognize and eradicate tumor cells while reducing resistance and suppressing their escape mechanisms (7, 8).

This review article discusses the rationale and mechanisms, as well as preclinical and clinical evidence behind combination immunotherapies for NSCLC treatment. It also summarizes the challenges associated with these regimens, such as managing increased toxicity and recognizing the right patient populations. By critically assessing the impact of combination immunotherapies, this review aims to provide insights that may redesign the NSCLC treatment setting, offering new hope for patients and clinicians tackling this life-threatening malignancy.

## 2 Non-small cell lung cancer

NSCLC is the most common type of lung-associated malignancy, accounting for approximately 85% of all lung cancer cases. NSCLC is a malignant condition that arises from the out-of-control growth of irregular cells in the lung tissue, and it comprises a diverse group of malignancies that share restricted features (9). Therefore, understanding different aspects of NSCLC pathogenesis is necessary for healthcare professionals, as it is essential in diagnosis, prognosis, and treatment. NSCLC is categorized into other subtypes with diverse characteristics and treatment-specific tactics. The principal subtypes are adenocarcinoma, squamous, and large-cell carcinoma (10). These subtypes are discriminated according to the appearance of the tumor cells under a microscope, the specific involved proteins, and genetic mutations (11). The leading cause of NSCLC is carcinogenic exposure, with cigarette smoking being the prominent risk factor (12). Other risk factors that can increase NSCLC risk include exposure to secondhand smoke, environmental toxins such as asbestos and radon, genetic predisposition, and lung-associated disorders (12–14) (**Figure 1**).

The most regular NSCLC symptoms may include a persistent cough, shortness of breath, chest pain, unexplained weight loss, coughing up blood, and recurrent lung infections (15, 16) (**Figure 1**). Early-stage NSCLC is often asymptomatic; therefore, routine screenings and early detection of the malignancy are critical for improved diagnosis and treatment processes. Diagnosing NSCLC classically involves a combination of imaging methods, such as X-rays, computed tomography (CT) scans, and magnetic resonance imaging (MRI) scans, as well as a biopsy for the microscopic assessment of lung tissue (17). Molecular evaluation is also critical to recognize the presence of specific genetic mutations or modifications that may guide treatment decisions (18). In addition, NSCLC staging is essential for determining tumor cell metastases and designing suitable disease management. The TNM system, which assesses primary tumor size, lymph node involvement, and distant metastases, is usually used for staging NSCLC. Stages range from 0 (localized to the lung) to IV (advanced, with distant metastases) (19). The prognosis for NSCLC varies widely and depends on the tumor stage at diagnosis, the specific subtype, the presence of genetic mutations, and the patient’s overall health (20). Early detection and advances in therapeutic approaches have improved survival rates, especially for those with localized disease. However, advanced-stage NSCLC remains challenging, and the overall prognosis could be more satisfactory.

NSCLC, as a diverse category of lung cancers, demonstrates discrete molecular subtypes that considerably influence treatment methods and prognoses (21). Specific genetic and molecular alterations can characterize these subtypes. Among them, mutations in the epidermal growth factor receptor (*EGFR*) gene are prevalent, predominantly in non-smokers and certain ethnic groups, guiding EGFR-targeted therapies. Anaplastic lymphoma kinase (*ALK*) and c-ros oncogene 1 (*ROS1*) rearrangements represent fusion events in their respective genes, provoking targeted therapies with *ALK* and *ROS1* inhibitors (22). Mutations in genes such as *BRAF*, *KRAS*, and human epidermal growth factor receptor 2 (*HER2*) also play crucial roles in subtyping NSCLC, with emerging targeted therapies tailored to these specific mutations (23) (**Figure 1**). Furthermore, assessing the expression of immune checkpoints, such as programmed death-ligand 1 (PD-L1), can estimate the possibility of response to ICIs (24). The landscape of NSCLC molecular subtypes continues to progress with ongoing investigations, necessitating regular updates to clinical practices for optimal disease management.

## 3 A brief look at conventional and targeted therapies, as well as immunotherapeutic approaches in NSCLC

Treating NSCLC depends on several factors, including cancer stage, histological subtype, and the patient’s overall health. Among conventional therapies for NSCLC, surgery is often recommended for patients with early-stage NSCLC (25). The aim is to remove the tumor tissue and potentially nearby lymph nodes (26). Radiation therapy may also be used as a primary treatment or in combination with surgery or chemotherapy, particularly in cases where surgery is not feasible (26). Chemotherapy is another commonly applied method in advanced NSCLC treatment to eliminate tumor cells or decrease their growth and proliferation (27). Targeted therapies and immunotherapy have also emerged as efficient opportunities in NSCLC cases with specific genetic mutations (28). Targeted therapies are drugs specifically targeting genetic mutations or modifications in the gene of tumor cell receptors or molecules, such as *EGFR*, *ALK*, and *ROS1* mutations (29). These therapies offer the potential for more personalized and effective treatment. Furthermore, ICIs like pembrolizumab and nivolumab have revealed promising outcomes in treating NSCLC by boosting their immune system responses against tumor cells (30) (**Figure 1**).

The neoadjuvant approach in cancer treatment offers several distinct advantages, particularly in the context of resectable NSCLC (31). Initiating treatment before surgery allows for prompt intervention, enhances patient compliance, and enables a detailed pathological assessment of the treatment’s effectiveness. This assessment is crucial, as it informs subsequent adjuvant therapies and aids in eradicating micro-metastatic disease (32). In recent years, evidence has supported the efficacy of neoadjuvant immunotherapy combined with chemotherapy (with or without adjuvant immunotherapy post-surgery) in resectable NSCLC. Trials such as NADIM II and CheckMate 816 have demonstrated significant improvements in pathological complete response (pCR) rates and progression-free survival (PFS) or event-free survival (EFS) when utilizing neoadjuvant immunotherapy–chemotherapy combinations (33, 34). The Keynote 671 trial has further bolstered these findings by showcasing enhancements in EFS alongside overall survival (OS) benefits (35, 36). Similarly, studies like CheckMate 77T and AEGEAN have reported similar positive outcomes, although the data regarding OS are still maturing (37, 38). These advancements indicate the increasing importance of neoadjuvant strategies, particularly incorporating immunotherapy, in the multidisciplinary management of resectable NSCLC.

Tyrosine kinase inhibitors (TKIs) have become integral to the targeted therapy landscape for NSCLC, offering a personalized approach based on the molecular characteristics of patients’ tumors. For instance, in *EGFR* mutations, first-generation TKIs like erlotinib, gefitinib, and third-generation osimertinib have shown efficacy, especially in non-smokers and specific ethnic populations (39).

As briefly discussed in this section, several immunotherapeutic approaches for NSCLC act through various mechanisms to reinforce the immune system to combat tumor cells.

### 3.1 Immune checkpoint inhibition

Among immunotherapeutic approaches, ICIs are monoclonal antibodies against cytotoxic T lymphocyte antigen 4 (CTLA-4), programmed cell death protein-1 (PD-1), and PD-L1 inhibitory molecules (20) (**Figure 1**). Tumor cells express these inhibitory molecules to evade immune surveillance by suppressing antitumor immune responses, such as CD8^+^-mediated tumor cell killing (40). ICIs disrupt the interaction between effector immune cells and tumor or immunosuppressive cells, inducing antitumor responses (41). Drugs like pembrolizumab, nivolumab, atezolizumab, and other ICIs have been used for NSCLC treatment (42). Nivolumab, the first humanized monoclonal antibody against PD-1, demonstrated promising clinical outcomes in trials (43). Both CheckMate 057 and CheckMate 017 studies reported prolonged OS and a better safety profile than docetaxel, particularly in NSCLC patients with higher PD-L1 expression (44, 45). The pooled analysis of these studies reiterated these benefits, illuminating lower rates of adverse events (AEs) and increased OS with nivolumab. However, a study involving previously untreated NSCLC patients with 5% or more PD-L1 expression showed no significant difference in OS and PFS compared to chemotherapy despite a better safety profile (46).

Pembrolizumab, another PD-1 inhibitor, demonstrated its efficacy in KEYNOTE-001, proving durable antitumor activity and high 5-year OS rates, especially in treatment-naïve patients and those with higher PD-L1 expression (47). KEYNOTE-024 established pembrolizumab as a first-line treatment option, significantly improving PFS and objective response rates in patients with high PD-L1 expression (48). This led to Food and Drug Administration (FDA) approval for newly diagnosed advanced NSCLC patients with a PD-L1 expression rate of 50% or more. Atezolizumab, targeting PD-L1, showed promising efficacy in different trials, particularly in the first-line setting, improving OS in NSCLC patients with high PD-L1 expression, leading to FDA approvals after post-chemotherapy progression and as a first-line treatment in patients with high PD-L1 expression (49, 50). Avelumab demonstrated potential benefits in JAVELIN Lung 200, mainly in subgroups with higher PD-L1 expression, despite initially failing to prolong OS significantly versus docetaxel. The 2-year follow-up showed promising clinical outcomes, doubling the 2-year OS rates in specific PD-L1 subgroups (50, 51). Durvalumab, after chemoradiotherapy, significantly improved OS compared to the placebo in stage III NSCLC patients, leading to its approval for this population by the FDA. Long-term follow-up results confirmed the durable PFS and prolonged OS benefits of durvalumab (52, 53). Cemiplimab, a PD-1 inhibitor, exhibited substantial enhancements in OS and PFS compared to chemotherapy in advanced NSCLC patients with PD-L1 expression of at least 50%, offering a potential novel treatment for these patients (52, 54). The emergence of these immunotherapies has transformed NSCLC treatment, particularly in specific patient subsets with high PD-L1 expression, specifying hopeful opportunities for prolonged survival and a new direction in NSCLC management. Nonetheless, challenges persist in identifying broader beneficiary populations and increasing response rates, especially in monotherapy settings.

### 3.2 Tumor-infiltrating lymphocytes

Immunotherapy stimulates the infiltration of effector immune cells, particularly tumor-infiltrating lymphocytes (TILs), into the TME. Following the recruitment of TILs into the tumor milieu can recognize and eliminate tumor cells. Adoptive cell transfer (ACT) techniques involve isolating, expanding, and reinfusing TILs into patients, enhancing the antitumor immune response (55). There have been few extensive studies on TIL therapy for NSCLC; despite the potential responsiveness of this malignancy to PD-1/PD-L1 inhibitors and its high mutational burden, TIL therapy may be an appropriate therapeutic option. The initial clinical study of TIL therapy in NSCLC, published in 1996, successfully expanded TIL cultures from tumors removed during standard surgical procedures in 113 out of 118 patients with stage II and III lung cancer (56). The patients were randomly assigned to receive TIL therapy alone for stage II or in combination with standard chemoradiotherapy for stage III. TIL expansion to substantial numbers was accomplished using high doses of interleukin-2 (IL-2), and these cells were infused without any preconditioning regimen. Subsequent research indicated that preconditioning significantly improves the effectiveness of TIL therapy by creating space and nutrients for TIL expansion while limiting the negative impact of Tregs and MDSCs (57). Another study in this context reported that despite limitations, a significant improvement in 3-year OS was observed, particularly in stage III patients, compared to the control group.

Furthermore, a noticeable decrease in local relapse was evident in this group. The study highlighted the most substantial benefits of adding TIL therapy within 6 months post-treatment. This observation may suggest a limited persistence of the transferred cells, potential exhaustion of the infused T cells due to high IL-2 doses during expansion, or their suppression by Tregs and MDSCs (58). Nevertheless, this study laid the foundation by demonstrating that TIL therapy could be effectively applied to patients in advanced stages of lung cancer despite encountering challenges and limitations. The findings signify the potential efficacy in survival and reducing relapse, establishing a fundamental understanding of TIL therapy’s potential in treating late-stage lung cancer (58).

### 3.3 Cytokine therapy

Cytokines are small signaling glycoproteins that regulate immune responses (59). IL-2 and interferons are the most important examples of cytokines used in cancer immunotherapy (60, 61). IL-2 therapy aims to stimulate the proliferation and activation of T cells, boosting their ability to recognize and attack cancer cells (62). Interferons modulate the immune response, potentially reinforcing the recognition and destruction of cancer cells (63). In a clinical investigation, NSCLC patients underwent a therapeutic regimen employing a combination of IL-2 and interferon-alpha (IFN-α) at two distinct dosage levels (64). This treatment regimen included the administration of specified doses via intravenous and intramuscular routes over defined periods, interspersed with designated rest intervals. Several adverse effects, notably anorexia, fatigue, nausea, and headaches, were recognized during the treatment; however, these effects did not reach a severity necessitating a reduction in dosage. Despite administering this regimen to 11 participants, there were no noticeable positive responses. Distressingly, nine of the 11 patients experienced disease progression within 5 weeks of commencing the treatment.

Consequently, the investigation ultimately determined that the therapeutic approach involving the combined use of IL-2 and IFN-α exhibited more ineffectiveness in addressing the conditions presented by the NSCLC patients (64). It has been demonstrated that IL-6 plays an essential role in response to injury or infection and is a promising biomarker for predicting poor prognosis and therapeutic targets in NSCLC. Studies have shown that anti-IL-6 therapy could not directly affect the effect of ICIs but could enhance their anticancer function, which may be an option for managing immune-related adverse events (irAEs) and a therapeutic target for treating NSCLC (65).

It has been revealed that IL-6 plays a significant role in inhibiting the phosphoinositide 3-kinase (PI3K)/Akt/mammalian target of the rapamycin (mTOR) pathway, which is essential for regulating cellular growth, proliferation, and metabolism (66). This inhibition impacts primary cellular energetic and anabolic processes, disrupting normal cellular function and homeostasis​​. Furthermore, IL-6 exerts systemic effects by altering various metabolic processes, including energy, protein, lipid, and glucose metabolism (67). It contributes to insulin resistance, promotes lipolysis, and facilitates the mobilization of free fatty acids​​. These systemic changes can profoundly affect metabolic health and disease states (68). IL-6 is also a key mediator in cancer-related conditions such as anemia and anorexia, which significantly impair the nutritional intake of essential substrates and microelements like glucose, iron, and zinc (66, 69). These nutrients are vital for optimal lymphocyte activity and overall immune function​​.

Growing evidence indicates that cachexia, a complex syndrome caused by cancer-related chronic inflammation commonly seen in patients with NSCLC, can weaken the immune response and reduce the effectiveness of ICIs (70). Moreover, the inflammatory response driven by IL-6 is strongly correlated with elevated levels of C-reactive protein (CRP) and other acute-phase proteins, like fibrinogen. This occurs through direct transcriptional induction in the liver, which is dose- and time-dependent. Acute-phase proteins, in turn, contribute to immunosuppression and are associated with leukocytosis and lymphopenia alongside IL-6. Elevated inflammatory markers, such as CRP and the neutrophil-to-lymphocyte ratio (NLR), have been significantly correlated with cancer cachexia and sarcopenia (66, 71, 72). These biomarkers highlight the inflammatory state associated with cachexia and underscore the systemic impact of IL-6-driven inflammation on patients’ overall health status (70). In contrast, analyzing a dataset of various morphological stages in lung squamous cell carcinoma development obtained from cancer patient biopsies revealed that the adaptive immune response is strongest at the earliest stages. In contrast, the most advanced invasive stages are marked by increased expression of co-inhibitory molecules and suppressive cytokines, such as PD-L1, IL-10, and IL-6 (73).

The challenge lies in using high doses of cytokines necessary to provoke a significant anticancer response. These high doses have drawbacks, including the cytokines’ short lifespan within the body and the risk of systemic toxicity, leading to pro-inflammatory and autoimmune reactions (74).

### 3.4 Cancer vaccines

Vaccines designed to trigger an immune response against tumor-specific antigens have been under development. These vaccines, which may contain tumor-specific antigens (TSAs), peptides, or DNA, aim to activate the immune system, recognizing and attacking tumor cells (75). However, their effectiveness in NSCLC is still under investigation. Certain cancer patients exhibit limited benefits from immunotherapy, particularly those with a low response rate, a phenomenon often associated with a restricted T-cell response against tumor cells, especially in cases of tumors with a low mutational burden (76). The effectiveness of immunotherapy hinges on the presence of preexisting intratumoral CD8^+^ T cells, highlighting the need to induce cytotoxic CD8^+^ T cells through vaccination (77). Cancer vaccines can generate tumor-specific T cells in the periphery or within the tumor tissue. They can also facilitate the migration of activated peripheral T cells into the TME, augmenting the presence of TILs (78).

Furthermore, cancer vaccines induce tumor cell death, releasing tumor antigens (antigen cascade or epitope spreading) and initiating more robust tumor-specific immune responses (79).

In contrast to ICIs, which enhance inactivated responses of effector T cells, vaccination can activate tumor-specific naïve T cells, broadening the spectrum of tumor-specific immune responses (80). As a result, combining cancer vaccines with ICIs can prompt specific T-cell responses, which is proposed as an attractive therapeutic option to enhance the overall efficacy of immunotherapy for cancer patients (81). Evidence indicates a growing interest in cancer vaccines as a possible adjunct in treating and preventing NSCLC. These vaccines, including CIMAvax-EGF and other vaccines against MAGE-A3 and hTERT, have shown varying degrees of safety, immunogenicity, and some improvements in managing NSCLC. Ongoing and future clinical trials are essential to understand their efficacy further, optimize combinations, and explore their roles in diverse stages and risk groups of NSCLC (81).

### 3.5 Chimeric antigen receptor T-cell therapy

Ongoing research aims to identify biomarkers that predict patient responses and improve treatment strategies to combat resistance mechanisms in NSCLC immunotherapy (82). In solid tumors, T lymphocytes have been modified using synthetic chimeric antigen receptors (CARs) to target specific tumor-associated antigens (TAAs) found in various human malignant tumors, with a significant focus on NSCLC (83, 84). Within NSCLC, the most commonly targeted antigens encompass EGFR, mesothelin (MSLN), mucin 1 (MUC1), prostate stem cell antigen (PSCA), carcinoembryonic antigen (CEA), PD-L1, CD80/CD86, inactive tyrosine-protein kinase transmembrane receptor (ROR1), and human EGFR2 (82). In this regard, a study showed that recombinant anti-EGFR CAR-T cells exhibit specific cytolytic activity against EGFR-positive tumor cells and can release cytokines, displaying potential in fighting NSCLC (85). Ongoing clinical trials assessed the safety and efficacy of different approaches using modified anti-EGFR CAR-T cells and demonstrated partial patient responses and stability (86). However, clinical data regarding anti-HER2 CAR-T-cell therapy for NSCLC remains pending due to ongoing investigations and adjustments in the trial structure for safety considerations (87). These data indicate the diverse landscape of CAR-T-cell therapy targets for NSCLC, showing varied stages of development and promising results in preclinical and clinical settings. Further clinical trials are warranted to confirm the safety and efficacy of these therapies, potentially opening new avenues for treating NSCLC with precision immunotherapy (82).

## 4 Rationale for combination immunotherapies

The rationale for combination immunotherapeutic methods in NSCLC stems from the complexity and heterogeneity of the TME, the diverse mechanisms of immune evasion used by tumor cells, and the limitations observed in mono-immunotherapy (88, 89). Combining different immunotherapeutic tactics or other antitumor agents aims to stimulate antitumor immune responses, broaden the scope of antitumor activity, and hypothetically overcome resistance mechanisms, eventually improving treatment outcomes for NSCLC patients (90, 91).

**Figure 2:**
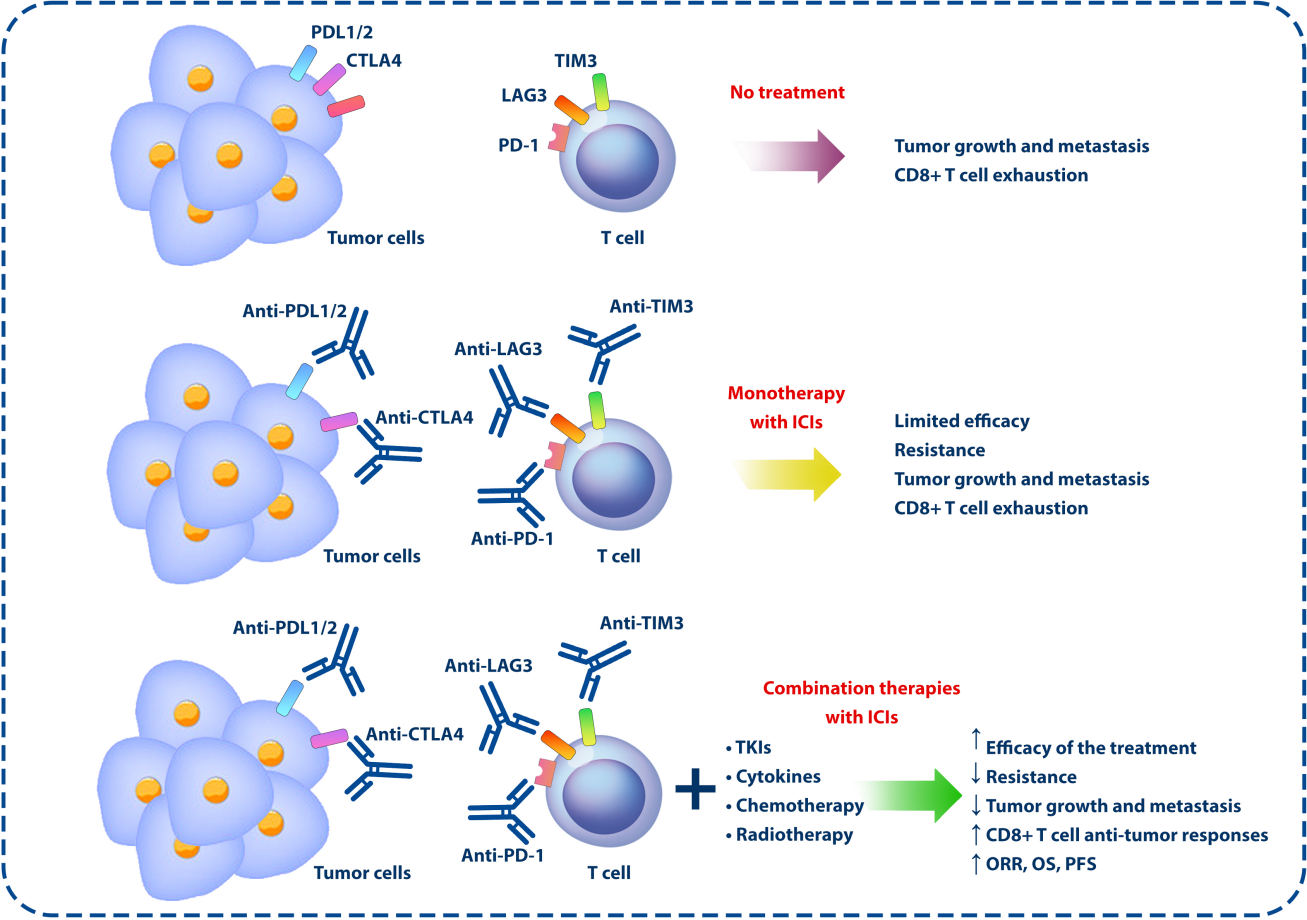
Monotherapies and combination therapies using ICIs and other anticancer approaches. In the TME, the expression of inhibitory molecules on the surface of immune and tumor cells can lead to tumor development and growth by exhausting CD8^+^ T cells and inhibiting the antitumor responses of the immune system. However, according to the studies conducted in this field, the use of ICIs alone cannot have a significant effect in the treatment of cancer, but by combining these inhibitors with other anticancer agents, such as chemotherapy, radiotherapy, TKIs, and cytokines, the effectiveness of the treatment can be increased. ICIs, immune checkpoint inhibitors; TME, tumor microenvironment; TKIs, tyrosine kinase inhibitors.

NSCLC tumors show considerable heterogeneity, not only in their genetic makeup but also in their interaction with the immune system (92). In addition, tumor cells use multiple mechanisms to escape immune surveillance, such as increasing the expression of immune checkpoint molecules, releasing immunosuppressive factors, and modulating antigen presentation (93). In addition to recognizing and responding to TAAs, CD8^+^ T cells release pro-inflammatory cytokines that allow them to mount a pro-inflammatory response and eliminate tumor cells (94). A lower progression rate and longer survival rate are observed in cancer patients with more extensive intratumor infiltration of CD8^+^ T cells, demonstrating the importance of CD8^+^ T cells in antitumor immunity (95). However, most CD8^+^ TILs display various degrees of dysfunction, including low proliferation, impaired cytokine production, and inability to lyse target cells (96, 97). An increase in inhibitory checkpoint molecules, such as PD-1, T-cell immunoglobulin mucin-3 (TIM-3), lymphocyte activation gene-3 (LAG-3, CD223), T-cell immunoreceptor with Ig and ITIM domains (TIGIT), and B- and T-lymphocyte attenuator (BTLA), on CD8^+^ TILs and other cells in the TME explains these functional defects and suppressing antitumor immune responses. Therefore, suppressing these molecules using combinations of ICIs can effectively slow tumor growth, promote tumor regression, and extend survival in humans with cancer (97, 98) (**Figure 2**).

Evidence reveals that there should be more than single-agent immunotherapies to tackle all these evasion strategies. Combining different ICIs targeting various checkpoints (e.g., PD-1, PD-L1, and CTLA-4) has been investigated to overcome resistance and broaden the activation of immune responses (99). Concurrently hindering multiple immune checkpoints can potentially enhance the activation of T cells and improve their ability to recognize and attack cancer cells (100). Different immunotherapies have distinct mechanisms of action. For instance, combining a checkpoint inhibitor with an agent such as IL-2 that stimulates T-cell activation may complement each other, simultaneously tackling multiple aspects of immune evasion (101). Some patients do not respond to mono-immunotherapy or develop resistance mechanisms over time (102, 103). Combination therapies aim to overcome or delay resistance by targeting multiple pathways, potentially improving response rates and extending the duration of response (DOR) (104).

Combining different immunotherapies may lead to a broader and more robust immune response against cancer cells, potentially affecting tumor growth and spreading more effectively (104). It should be noted that these combination therapies can also lead to increased toxicity, as observed in previous studies that combined ICIs with other anticancer agents (105, 106). Managing adverse effects and determining the optimal dosing and scheduling are essential considerations. Ongoing challenges are selecting the most effective combinations and identifying biomarkers that predict patient response to these combinations (107). Personalizing treatment based on these biomarkers remains a critical aspect of combination immunotherapy (108). Conducting robust clinical trials to assess safety and efficacy is crucial in gaining regulatory approval for combination therapies.

Collectively, the rationale for combining immunotherapies in NSCLC is primarily grounded in addressing mono-immunotherapies’ limitations, overcoming immune evasion mechanisms, and enhancing the antitumor immune response. While promising, the optimal combination therapies with maximized efficacy and minimized toxicity remain areas of active research and clinical investigation.

## 5 Immunotherapeutic agents in combination therapies

As discussed, ICIs play a significant role in treating patients with NSCLC; however, combining these agents with other therapeutic approaches is ongoing due to their limitations in monotherapies. Regarding the limited efficacy of monotherapy using ICIs, preclinical research has revealed that neoadjuvant ICIs outperform adjuvant ICIs in eliminating distant metastases, owing to a sustained and intensified tumor-specific immune response (109). Moreover, experiments in murine models indicate that combined neoadjuvant immunotherapy displays greater efficacy than the adjuvant approach, hinting at the potential for more advanced and robust treatment strategies (110) (**Figure 2**). This section discusses various combination therapies in NSCLC, focusing on immunotherapeutic, targeted, and conventional therapies.

### 5.1 Combining immune checkpoint blockers and conventional therapies

In treating locally advanced unresectable NSCLC, administrating durvalumab, a PD-L1 inhibitor, has demonstrated a remarkable benefit in OS as a consolidation therapy post-chemoradiotherapy, as evidenced by the phase III PACIFIC trial where approximately half of the treated patients remained alive at the 4-year mark (53, 111). Several phase III trials are currently underway to investigate the efficiency of immunotherapy in the adjuvant setting. These trials investigate utilizing PD-1 or PD-L1 alone or combined with concurrent chemotherapy (112). For instance, IMpower010 disclosed a disease-free survival (DFS) advantage in the atezolizumab arm, particularly noticeable among patients whose tumors showed high PD-L1 expression (>50%) (113). Notably, patients in this trial primarily presented with locally advanced disease and were required to undergo chemotherapy per the trial’s protocol. Meanwhile, the Keynote 091 trial examining adjuvant pembrolizumab in stage IB-IIIA NSCLC shows potential for increasing DFS, as per a preliminary press release, although specific results are not yet accessible. Both trials eagerly await OS results, but it remains to be seen if, similar to atezolizumab, only patients with PD-L1 > 50% will benefit from DFS. Despite their compelling nature, the ICIs may be more biologically interesting as a neoadjuvant strategy. With maximal exposure to tumor neoantigens during treatment in this context, the patient will gain a sustained immunologic memory (114). A retrospective analysis focused on understanding the efficacy of ICI in NSCLC, explicitly concerning the *KRAS* mutational status (115). Drawing on data from 12 registrational clinical trials investigating first-line (1L) ICI treatment, either alone or in combination with chemotherapy, the study included 1,430 patients, with 61% exhibiting wild-type *KRAS* and 39% harboring *KRAS* mutations. Notably, the *KRAS G12C* mutation was identified in 11% of the *KRAS*-mutated subset. Patient demographics, such as gender, ethnicity, PD-L1 expression, and smoking history, exhibited similarity across *KRAS*-mutated, *G12C*, and wild-type groups. The key revelation from this pooled analysis is that patients with *KRAS*-mutated NSCLC, including the specific *G12C* mutation, appear to derive substantial benefit from first-line chemo-ICI, comparable to those with *KRAS* wild-type NSCLC. Combining chemotherapy with ICI demonstrated superior efficacy than ICI or chemotherapy alone in *KRAS*-mutated patients. However, a relatively small number of patients constrain the interpretation for the subgroup with the documented *KRAS G12C* mutation. Therefore, this study recommends adding a chemo-ICI comparator arm to clinical trials investigating targeted therapies for *KRAS*-mutated NSCLC (115).

A retrospective study aimed to identify the optimal initial treatment for patients with NSCLC and malignant pleural effusion (MPE) undergoing ICI therapy. The analysis included patients who received a combination of ICI and chemotherapy (ICI/Chemo) or pembrolizumab monotherapy (116). Propensity score matching (PSM) was employed to minimize potential biases. In the PD-L1 high cohort (143 patients), after PSM, ICI/Chemo exhibited a significantly prolonged median PFS compared to pembrolizumab monotherapy (11.1 versus 3.9 months, respectively; p = 0.0409). In the ICI/Chemo cohort (139 patients), wherein some regimens featured bevacizumab (BEV), known for MPE control, 23 patients received BEV. PSM analysis revealed no significant difference in median PFS between the BEV and non-BEV groups (6.1 versus 7.4 months; p = 0.9610). The study suggests that ICI/Chemo may be more effective than pembrolizumab monotherapy for non-squamous NSCLC patients with MPE. However, the synergistic impact of BEV with ICI/Chemo appears limited, underscoring the necessity for further investigations into the critical factors influencing tumor-induced immunosuppression in this specific patient population (116).

Another study aimed to explore the practical effectiveness and safety of combined cytotoxic chemotherapy and PD-1/PD-L1 inhibitor therapy for advanced NSCLC, explicitly focusing on individuals aged 75 or older and those with a performance status of 2 or higher (117). Analyzing a cohort of 299 chemo-naïve patients who underwent treatment with a combination of platinum, pemetrexed, and pembrolizumab, the findings revealed a significant association between better performance status (0–1) and a higher PD-L1 tumor proportion score (≥50%) with improved PFS. Notably, the real-world efficacy of the combination therapy was limited in patients with poor performance status. Furthermore, the study identified a heightened occurrence of severe AEs in older people and individuals with poor performance status compared to their younger and healthier counterparts. A remarkable detection was the substantial number of patients experiencing AEs leading to treatment termination, with a notably higher rate in older patients. Hence, physicians are advised to exercise caution, especially when considering this regimen for elderly and poor-performance status patients, emphasizing that a balanced evaluation of potential benefits and risks is imperative (117). A study aimed to assess how first-line pembrolizumab, in combination with pemetrexed and carboplatin, performed for patients with metastatic NSCLC (mNSCLC) in real-world scenarios outside clinical trial settings (118). Researchers used data from a deidentified US electronic health record-derived database, focusing on 377 eligible patients without prior systemic anticancer therapy without specific genetic alterations (*EGFR/ALK/ROS1*). The patients with good performance statuses began pembrolizumab-combination therapy between May 2017 and January 2019, excluding those involved in clinical trials. The findings indicated a median OS of 17.2 months, a median treatment duration of 5.8 months for pembrolizumab, and a real-world response rate of 39.3%. Survival rates at 12 and 24 months varied across patients with different PD-L1 expression levels, with higher PD-L1 expression associated with somewhat more promising outcomes. The median real-world PFS was 6.2 months, while the median DOR reached 13.1 months. These outcomes indicate the favorable effects of first-line pembrolizumab-combination therapy for mNSCLC patients with wild-type *EGFR/ALK*, emphasizing the observed benefits in real-world settings at US community oncology clinics (118).

Another study aimed to assess the efficacy of ICI-based treatments compared to traditional chemotherapy for patients with mNSCLC who developed resistance to EGFR-TKIs (119). The cohort comprised 132 patients from two cancer centers in China, with a median follow-up time of 21.7 months from the onset of EGFR-TKI resistance. The results revealed a median PFS of 4.9 months and an OS of 13.5 months. Multivariate analysis, adjusting for gender, age, mutation status, and metastasis to the brain or liver, demonstrated that ICI-based therapy significantly improved OS compared to classical chemotherapy [hazard ratio (HR), 0.55; 95% confidence interval (CI), 0.34–0.88; p = 0.01]. The combination of ICI and chemotherapy showed a sustained OS benefit across various subgroups, including younger patients (<65 years), those without secondary *T790M* mutations, and individuals without liver and brain metastases, particularly those with good Eastern Cooperative Oncology Group (ECOG) scores. Accordingly, for patients with EGFR-TKI resistance, ICI-based therapy, especially in combination with chemotherapy, exhibited superior survival outcomes compared to traditional chemotherapy, suggesting its potential as a preferred treatment approach, particularly in this patient subgroup (119). Another study addressed the lack of evidence regarding the efficacy and safety of ICIs in patients with NSCLC harboring *EGFR* mutations who have experienced resistance to EGFR TKIs (120). Clinical data from real-world settings were collected, and a time series-based meta-analysis was conducted. The study included 22 NSCLC patients from two hospitals with *EGFR* mutations after TKI resistance. The median PFS for these patients was 5.6 months. When stratified by treatment strategy, the median PFS was 2.4 months for the ICI monotherapy group and 5.9 months for the ICI combined chemotherapy group.

Additionally, a broader analysis incorporating 16 studies, comprising five trials, 10 controlled cohorts, and one real-world study, evaluated ICI-treated NSCLC patients with *EGFR* mutations after TKI failure. The 6-month survival rate was 0.82 (95% CI, 0.36–0.97), and the PFS rate was 0.55 (95% CI, 0.34–0.74). ICI combined with chemotherapy demonstrated the best survival outcome, as indicated by the 12-month survival rate and PFS. No new safety signals were identified with combination therapy, and the frequency of treatment-related AEs was similar to that reported in previous studies of chemotherapy combined with ICIs. The findings suggest that combining ICIs with chemotherapy may significantly improve PFS among patients with locally advanced or metastatic non-squamous NSCLC who have developed EGFR-TKI resistance (120).

A double-masked, randomized, phase 3 trial (CameL-sq, NCT03668496) is investigating the effectiveness and safety of camrelizumab, a humanized immunoglobulin G4-κ monoclonal antibody targeting PD-1 when combined with chemotherapy as a primary treatment for patients facing advanced squamous NSCLC. The study enrolled 389 patients with stage IIIB-IV squamous NSCLC who were randomly assigned to receive either camrelizumab plus chemotherapy or placebo plus chemotherapy. The treatment regimen involved four to six cycles of carboplatin plus paclitaxel, followed by maintenance therapy with camrelizumab or a placebo administered every 3 weeks. Results demonstrated that the combination of camrelizumab and chemotherapy significantly extended PFS (median 8.5 months vs. 4.9 months) and OS (median not reached vs. 14.5 months) compared to the placebo-chemotherapy group (p < 0.0001 for both). No unexpected irAEs were observed in either treatment group. Notably, biomarker analysis focused on circulating tumor DNA (ctDNA) dynamics, revealing that ctDNA clearance after two treatment cycles was independently associated with considerably longer PFS and OS in the camrelizumab plus chemotherapy group. These findings suggest that the combination of camrelizumab and chemotherapy stands out as a promising first-line treatment option for advanced squamous NSCLC, and the dynamics of on-treatment ctDNA may serve as a potent predictor for the efficacy of this combined therapeutic approach (121).

The KEYNOTE-789 study is a randomized, double-blind, phase 3 trial evaluating the efficacy of adding pembrolizumab to chemotherapy in patients with TKI-resistant, *EGFR*-mutant, metastatic non-squamous NSCLC (122). The study enrolled 492 randomized patients to receive pembrolizumab with chemotherapy (pembro + chemo) or placebo with chemotherapy (pbo + chemo). The primary endpoints were PFS and OS. At the second interim analysis (IA2), the median PFS with pembrolizumab with chemotherapy was 5.6 months compared to 5.5 months with placebo with chemotherapy, and the results did not reach statistical significance. The final analysis (FA) at a data cutoff on January 17, 2023, showed a median OS of 15.9 months with pembrolizumab with chemotherapy versus 14.7 months with placebo with chemotherapy. Although the HR favored pembro + chemo for OS (0.84), it did not reach statistical significance. The objective response rate (ORR) was similar between the two groups, and the DOR was also comparable. Grade ≥ 3 treatment-related AEs occurred in 43.7% of patients in the pembro + chemo arm and 38.6% in the pbo + chemo arm. Immune-mediated AEs and infusion reactions were higher in the pembro + chemo arm but were generally manageable. No new safety signals were identified. In conclusion, the addition of pembrolizumab to chemotherapy did not significantly prolong PFS and OS compared to placebo + chemo in patients with TKI-resistant, EGFR-mutant, metastatic non-squamous NSCLC in the KEYNOTE-789 study. AEs were manageable in both arms, and there were no new safety concerns (122).

The PEMBRO-RT trial sought to enhance the efficacy of immunotherapy in advanced-stage NSCLC by combining pembrolizumab with stereotactic body radiation therapy (SBRT). The study investigated immune infiltrates within the TME to understand the effects of this combination strategy (123). Tumor biopsies from patients treated with pembrolizumab alone or in combination with SBRT were analyzed at baseline and during treatment using multiplex immunofluorescence. CD3, CD8, CD20, CD103, and forkhead box P3 (FoxP3) were employed for lymphocytes and pan-cytokeratin for tumors, and human leucocyte antigen (HLA)-ABC expression was determined. Results revealed a significant increase in the total number of lymphocytes after 6 weeks of treatment in both the anti-PD-1 alone and anti-PD-1 + SBRT groups. The combination of SBRT and anti-PD-1 led to a substantial rise in CD103^+^ cytotoxic T cells. Notably, responders exhibited a higher baseline lymphocyte count compared to non-responders. This exploratory study suggests that overall lymphocyte infiltration, rather than a specific subset, is associated with a favorable therapeutic response in NSCLC patients undergoing this combined immunotherapy and radiation approach (123). According to recent emerging evidence, SBRT kills tumor cells directly and destroys tumor vascular beds, indirectly deteriorating the intra-TME and killing tumor cells. SBRT stimulates antitumor immunity by releasing large amounts of tumor antigens directly and indirectly, suppressing tumor recurrence and metastatic spread (124).

A randomized, double-blind, phase III study investigated the efficacy and safety of the ipilimumab + paclitaxel + carboplatin combination in patients with advanced NSCLC (125). The trial enrolled chemotherapy-naïve patients with stage IV or recurrent squamous NSCLC. The primary endpoint was OS, and the investigation also evaluated PFS and safety parameters. The findings showed that this combination therapy did not considerably prolong OS compared to chemotherapy alone, with a median OS of 13.4 months for chemotherapy plus ipilimumab and 12.4 months for chemotherapy plus placebo. PFS was almost equal in both groups. The safety profile showed higher rates of grade 3 or 4 treatment-related AEs with chemotherapy plus ipilimumab than chemotherapy plus placebo. Chemotherapy plus ipilimumab was associated with seven treatment-related deaths and chemotherapy plus placebo with one. Accordingly, in advanced squamous NSCLC, ipilimumab + chemotherapy does not improve survival, and ongoing studies assess the potential of nivolumab in combination with ipilimumab (125).

### 5.2 Combining different immune checkpoint blockers together

The CheckMate 227 trial reported compelling outcomes on the long-term efficacy and safety of first-line treatment with nivolumab plus ipilimumab in patients with advanced NSCLC (126). The median follow-up was 54.8 months, and the OS remained significantly longer with nivolumab plus ipilimumab than chemotherapy in both PD-L1 ≥1% and <1% subgroups. HRs of 0.76 and 0.64 for these subgroups indicate a significant decrease in the risk of death. Remarkably, the 4-year OS rates disclosed enduring advantages, with 29% versus 18% (PD-L1 ≥ 1%) and 24% versus 10% (PD-L1 < 1%) for nivolumab plus ipilimumab versus chemotherapy. The benefit of the treatment extended beyond histological classifications, encompassing both squamous and non-squamous NSCLC. The safety profile was consistent with previous studies, with rash being the most common irAEs. The study demonstrated that the early occurrence of irAEs was managed according to the guidelines. Despite the long-term benefits of nivolumab plus ipilimumab despite treatment-related AEs, this immunotherapeutic approach remains durable and effective at the 4-year mark (126).

The ARCTIC trial (NCT02352948) evaluated the safety and clinical outcome of administering durvalumab, an anti-PD-L1 antibody, as monotherapy and combined with tremelimumab in patients with advanced NSCLC (127). The trial recognized the potential limitations associated with PD-L1 monotherapies. It aimed to explore their efficacy in patients irrespective of their PD-L1 tumor status and design involved two sub-studies: Sub-study A focused on patients with PD-L1^+^ tumors (≥25% of tumor cells with membrane staining using VENTANA PD-L1 [SP263] CDx Assay) and evaluated the safety and clinical activity of durvalumab compared to standard of care (SoC) options such as erlotinib, gemcitabine, or vinorelbine. Sub-study B, in contrast, concentrated on patients with PD-L1^−^ tumors and assessed the combination of durvalumab and tremelimumab and each agent as monotherapy against SoC. Eligible patients for both sub-studies were those with locally advanced or mNSCLC (stage IIIB/IV), excluding specific genetic mutations, and who had received at least two prior systemic regimens, including one platinum-based chemotherapy regimen. Sub-study A revealed significant advancements in the treatment outcomes for heavily pretreated mNSCLC patients receiving durvalumab compared to the SoC. The median OS for those on durvalumab reached 11.7 months, notably surpassing the 6.8 months observed with the conventional SoC. The HR for mortality was 0.63. Additionally, the PFS improved with durvalumab, recording a median duration of 3.8 months in contrast to the 2.2 months seen in the SoC group, translating to an HR of 0.71. Furthermore, Sub-study A underscores a notable disparity in treatment-related grade 3/4 AEs, with a 9.7% incidence for durvalumab recipients compared to a substantially higher 44.4% for those on SoC. These findings suggest that durvalumab demonstrates clinically meaningful enhancements in both OS and PFS for heavily pretreated mNSCLC patients, particularly in cases where PD-L1 expression is 25% or more. The safety profile aligns with prior studies, affirming the potential efficacy of durvalumab in this patient population (127).

A non-randomized, open-label, phase 1b trial aimed to evaluate the safety and antitumor activity of combination therapy with durvalumab and tremelimumab in patients with advanced squamous or NSCLC (128). Immunotherapy-naïve patients with locally advanced or mNSCLC were enrolled in this trial. In the dose-escalation phase, 102 patients received different doses of durvalumab and tremelimumab. The primary endpoint was safety, and the maximum tolerated dose (MTD) was exceeded in the cohort receiving durvalumab 20 mg/kg every 4 weeks plus tremelimumab 3 mg/kg, with dose-limiting toxicities observed in 30% of patients in this group. The most common treatment-related grade 3 and 4 AEs included diarrhea, colitis, and increased lipase. Discontinuations due to treatment-related AEs occurred in 28% of patients, and severe treatment-related AEs occurred in 36%. Twenty-two patients died during the study, with three deaths related to treatment attributed to complications arising from myasthenia gravis, pericardial effusion, and neuromuscular disorder. Despite the observed adverse events, clinical activity was evidenced in patients with PD-L1^+^ and PD-L1^−^ tumors. ORR was achieved by 23% of patients in the combined tremelimumab 1 mg/kg cohort, with responses observed in both PD-L1^+^ and PD-L1^−^ tumors (128).

A phase 1 study (NCT02964013) focused on vibostolimab. This humanized antagonist monoclonal antibody blocks the interaction between the TIGIT and its ligands, CD112 and CD155 (129). In this investigation, vibostolimab monotherapy combined with pembrolizumab was well-tolerated, with a manageable safety profile across different doses in patients with advanced solid tumors during the dose-escalation/confirmation phase. The patients received vibostolimab (200 or 210 mg) plus pembrolizumab (200 mg) in 3-week cycles for up to 35 cycles. In the cohort of 41 anti-PD-1/PD-L1-naïve NSCLC patients, 73% received at least one prior line of therapy. Common treatment-related AEs included pruritus (34%), hypoalbuminemia (29%), and pyrexia (20%). Grade 3–4 treatment-related AEs occurred in 15% of patients, with no treatment-related AE deaths reported. The median duration of response was not reached, ranging from 4 to 17+ months. The combination of vibostolimab and pembrolizumab demonstrated promising antitumor activity and was well-tolerated in patients with advanced NSCLC who were naïve to anti-PD-1/PD-L1 therapy. These findings suggest the potential efficacy of this combination in a treatment-naïve NSCLC population, warranting further exploration in more extensive trials (129).

A phase I/II clinical trial aimed to explore the safety and efficacy of sabatolimab (anti-TIM3) monotherapy or in combination with spartalizumab (anti-PD-1) in patients with advanced solid tumors (130). The primary objectives of the phase I/Ib part were to evaluate safety and estimate the recommended phase II dose (RP2D) for future studies. Sabatolimab was administered intravenously in doses ranging from 20 to 1,200 mg every 2 or 4 weeks, while spartalizumab was given intravenously at doses ranging from 80 to 400 mg every 2 or 4 weeks. The MTD was not reached, and fatigue was the most common treatment-related AEs. Sabatolimab monotherapy did not show any responses, but when combined with spartalizumab, partial responses were observed in colorectal cancer, NSCLC, malignant perianal melanoma, and SCLC. These responses lasted between 12 and 27 months. Among the responsive patients, two had elevated expression of immune markers in baseline biopsies, and three had more than 10% TIM-3^+^ staining, including one patient with NSCLC who had received prior PD-1 therapy. The combination of sabatolimab and spartalizumab demonstrated promising tolerability and showed preliminary signs of antitumor activity. The RP2D for sabatolimab was determined as 800 mg every 4 weeks, with or without 400 mg spartalizumab every 4 weeks, suggesting a potential therapeutic option for patients with advanced solid tumors (130).

### 5.3 Combining immune checkpoint blockers and other anticancer approaches

Researchers explored the combination of anti-PD-1 and an adenovirus engineered to carry tumor necrosis factor-alpha (TNF-α) and IL-2 (Ad5-CMV-mTNFα/mIL-2) in a mouse NSCLC model (131). Despite the traditional use of local delivery in virotherapy, the treatment was administered intravenously to facilitate translation into clinical applications. This combination therapy notably reduced cancer growth in the animals, even in the presence of neutralizing antibodies. This reduction was associated with increased cytotoxic TILs, particularly the tumor-specific cells. This approach also decreased the immunosuppressive tumor-associated macrophage (TAM) population and improved dendritic cell (DC) maturation.

Additionally, the group that received anti-PD-1 in conjunction with the armed virus exhibited an expansion in the tumor-specific memory T cells within secondary lymphoid organs. However, the non-replicative nature of the Ad5-CMV-mTNFα/mIL-2 virus in the murine model raised concerns about its complete reflection of human clinical outcomes. To address this, the researchers complemented their findings using NSCLC *ex vivo* models that fully permitted the TNF-α and IL-2-armed oncolytic adenovirus TILT-123 activity. These findings highlight the potential of systemically administered adenovirus armed with TNF-α and IL-2 to enhance anti-PD-1 efficacy, emphasizing the necessity for further assessment in clinical trials (131).

Immune exhaustion and tumor growth are often caused by co-expression and upregulation of LAG-3 and PD-1 on T cells, and both pathways can be co-inhibited to improve CD8^+^ T cell antitumor responses (132, 133). A study aimed to address the limitations of immunoradiotherapy by combining NBTXR3-enhanced localized radiation with the simultaneous blockade of three different immune checkpoint receptors: PD-1, LAG-3, and TIGIT. This approach was tested in an anti-PD-1-resistant lung cancer mouse model. NBTXR3, a nanoparticle, was intratumorally injected into primary tumors, followed by localized radiation. Additionally, anti-PD-1, αLAG-3, and αTIGIT antibodies were administered intraperitoneally. The combination therapy effectively controlled the growth of both irradiated and distant unirradiated tumors, enhancing animal survival. Approximately 30% of treated mice experienced the destruction of both tumors. The treatment induced a robust activation of the immune response, characterized by increased numbers of immune cells and a transcriptional signature indicative of both innate and adaptive immunity within the tumors. Notably, mice treated with this combinatorial therapy demonstrated immunological memory responses when rechallenged with the same cancer cells, preventing tumor engraftment. This study supports the efficacy and validity of combining nanoparticle-enhanced radiotherapy with the simultaneous blockade of multiple immune checkpoint receptors. This approach may control tumor growth and induced immunological memory responses, highlighting its potential for translation into human patients. Further clinical investigations are required to explore the applicability and effectiveness of this combination therapy in cancer treatment (134).

Patients with advanced NSCLC-carrying activating *EGFR* mutations typically respond well to TKIs initially. However, resistance to these inhibitors often develops, driven by secondary *EGFR* mutations or EGFR-independent mechanisms like epithelial-to-mesenchymal transition (EMT). Unfortunately, post-EGFR-TKI resistance treatment options are limited, especially with anti-PD-1/PD-L1 inhibitors delivering minimal therapeutic benefit (135). Recognizing the association between IL-6 and poorer outcomes in NSCLC patients, a study aims to investigate whether IL-6 contributes to the immunosuppressed phenotype observed in these cases. The researchers employed a syngeneic genetically engineered mouse model (GEMM) of *EGFR*-mutant NSCLC to explore the impact of IL-6 on the TME and evaluate the combined efficacy of IL-6 inhibition and anti-PD-1 therapy. In parallel, *in vitro* studies utilized *EGFR*-mutant human cell lines and clinical specimens. The study identified that *EGFR-*mutant tumors exhibiting oncogene-independent acquired resistance to EGFR-TKIs displayed a more mesenchymal phenotype with significantly increased IL-6 secretion. In the *EGFR-*mutant GEMMs, depleting IL-6 enhanced the activation of infiltrating natural killer (NK) and T-cell subpopulations while reducing Tregs and Th17 cell populations. Inhibiting IL-6 also increased NK- and T cell-mediated killing of human osimertinib-resistant *EGFR*-mutant NSCLC tumor cells in cell culture. IL-6 blockade sensitized *EGFR*-mutant GEMM tumors to PD-1 inhibitors by increasing tumor-infiltrating IFNγ^+^ CD8^+^ T cells. These data suggest that IL-6 is upregulated in *EGFR*-mutant NSCLC tumors with acquired EGFR-TKI resistance, leading to suppressed T- and NK-cell function. Blocking IL-6 enhances antitumor immunity and the efficacy of anti-PD-1 therapy. The findings warrant further clinical investigations into combining IL-6 blockade with anti-PD-1 therapy (136).

The enduring clinical benefits of anti-PD-1 and anti-PD-L1 therapies in NSCLC are well-established; however, as discussed, patients possessing *EGFR* mutations within NSCLC demonstrate a comparatively diminished response to such treatments (137). The TME in NSCLC patients with *EGFR* mutations exhibits distinct characteristics significantly influencing the antitumor immune response (138). Activation of the EGFR pathway leads to increased PD-L1 expression in tumor cells, facilitating T-cell apoptosis and immune evasion (139, 140). EGFR-TKIs have been revealed to counteract these effects by enhancing MHC class I and II antigen presentation in response to IFN-γ, increasing levels of CD8^+^ T cells and DCs, reducing FOXP3^+^ Tregs, inhibiting the polarization of macrophages to the immunosuppressive M2 phenotype, and lowering PD-L1 expression on tumor cells. Accordingly, targeted therapies disrupt specific signaling pathways, while immunotherapies activate the immune system to target tumor cells that evade immune detection (138). The combination of TKIs and immunotherapy may yield suboptimal synergistic effects. An investigation reported that *EGFR*-mutated lung adenocarcinomas typically have a non-inflamed TME yet exhibit significant infiltration of CD4^+^ effector Tregs, which are more common in inflamed TMEs. EGFR signaling activates the cJun/cJun N-terminal kinase pathway, leading to increased CCL22, which recruits CD4^+^ Tregs. Concurrently, it reduces interferon regulatory factor-1 (IRF-1), resulting in decreased levels of CXCL10 and CCL5, which are crucial for CD8^+^ T-cell infiltration. The EGFR inhibitor erlotinib has been shown to reduce the infiltration of CD4^+^ effector Tregs in the TME. Moreover, combining erlotinib with anti-PD-1 could be more effective than monotherapies. These findings suggest that using EGFR inhibitors in combination with anti-PD-1 could enhance the effectiveness of cancer therapy in lung adenocarcinomas (141).

However, the data remain controversial in this context because active EGFR signaling enables NSCLC cells to deploy multiple strategies to create an immunosuppressive TME. These strategies include the recruitment of TAMs and Tregs, as well as the production of inhibitory cytokines and metabolites. To effectively overcome single-drug resistance, it is essential to characterize and target these mechanisms through a combined pharmacological approach. This approach should consider the disease stage, cancer-related inflammation, systemic symptoms, and the overall health status of the patient (138).

It has been found that purinergic signaling plays a crucial role in cancer progression and is regulated by nucleotidases. Several types of cancer have been found to overexpress CD73, the enzyme that breaks down AMP into adenosine, and various factors and mechanisms control CD73 expression (142). In contrast, the Ras-Raf-ERK pathway regulated CD73 expression directly through ERK1/2 without RSK or MSK involvement and was one of the downstream signaling pathways regulated by EGFR (143). An investigation showed that *EGFR*-mutated NSCLC manifests heightened CD73 expression in contrast to *EGFR* wild-type tumors, with CD73 expression under the regulatory influence of *EGFR* signaling (144). *EGFR*-mutated cell lines exhibit heightened resistance to T cell-mediated cytotoxicity, attributed to the curtailment of T-cell proliferation and function. In a xenograft mouse model representative of *EGFR*-mutated NSCLC, individual administration of either anti-PD-L1 or anti-CD73 antibodies fails to curtail tumor growth, in stark contrast to the isotype control. Conversely, the combined administration of both antibodies significantly inhibited tumor growth, amplified the presence of tumor-infiltrating CD8^+^ T cells, and augmented the production of IFN-γ and TNF-α by these T cells. Concurrently, a parallel elevation in gene expression is associated with inflammation and heightened T-cell function in tumors subjected to the combinatorial therapy of anti-PD-L1 and anti-CD73. These findings emphasize the rationale for combining anti-CD73 and anti-PD-L1 treatments as a promising therapeutic strategy for *EGFR*-mutated NSCLC, implicating an integral role for heightened T-cell activity in therapeutic response (144).

The positive clinical outcomes and tolerable safety profile of the frontline treatment regimen featuring camrelizumab, an anti-PD-1, in combination with low-dose apatinib, an angiogenesis inhibitor, has been revealed for previously untreated patients with advanced non-squamous NSCLC (145). Within the context of a multicenter, phase 1b and 2 study, this investigation focused on individuals characterized by a high tumor mutational burden (TMB) and the absence of *EGFR* or *ALK* alterations. The results showed a substantial ORR of 40.0%, with most patients achieving either partial responses or stable disease, leading to a high disease control rate of 92.0%. Notably, the median PFS of 9.6 months indicates a meaningful delay in disease advancement, while the median OS was not reached at the time of reporting, implying a potential for prolonged survival benefit. Remarkably, the positive clinical activity was consistent across patients irrespective of their PD-L1 expression levels. Although the safety profile was generally acceptable, common treatment-related grade 3 or higher AEs included increased gamma-glutamyl transferase, alanine aminotransferase, and abnormal hepatic function. These findings suggest that the combination therapy involving camrelizumab and low-dose apatinib has promising efficacy with manageable safety, presenting a potentially valuable therapeutic approach for advanced non-squamous NSCLC patients, regardless of their PD-L1 expression status (145).

Natural and genetically engineered viruses possess several antitumor mechanisms, making oncolytic viruses (OVs) an emerging therapeutic option for cancer. In addition to cytolysis, OVs potentiate the immune system by releasing antigens and activating inflammatory responses. Indirectly, OVs alter energy metabolism in tumor cells, modify the TME, and act as antiangiogenic agents (146). In this regard, the safety and efficacy of combining Teliso-V (telisotuzumab vedotin), an anti-c-MET-directed antibody–drug conjugate, with nivolumab was assessed in advanced NSCLC patients (147). While Teliso-V had previously demonstrated antitumor activity as a standalone therapy, its potential synergism with PD-1 inhibitors had not been explored. In this phase 1b study (NCT02099058), 37 adult patients with advanced NSCLC received varying doses of Teliso-V plus nivolumab. The primary focus was on safety and tolerability, with secondary objectives including the assessment of antitumor activity. As of January 2020, the efficacy analysis included 27 patients with c-Met immunohistochemistry-positive tumors. Notably, 74% of patients were treatment-naïve to ICIs, and the median age was 67 years. The most frequent any-grade treatment-related AEs included fatigue (27%) and peripheral sensory neuropathy (19%). The pharmacokinetic profile of Teliso-V plus nivolumab was found to be comparable to Teliso-V monotherapy. Despite favorable tolerability, the combination therapy exhibited limited antitumor activity, with an ORR of 7.4%. Two patients with PD-L1 positivity and a c-Met immunohistochemistry H-score of 190 and another with PD-L1 negativity and a c-Met H-score of 290 achieved confirmed partial responses. The overall median PFS was 7.2 months, with variations observed among different PD-L1 subgroups (PD-L1^+^: 7.2 months; PD-L1^−^: 4.5 months; PD-L1^unknown^: not reached). Consequently, Teliso-V and nivolumab combination was well tolerated in c-Met-positive NSCLC patients, although the observed antitumor activity was unassertive. These outcomes highlight the need for further investigation and exploration of alternative or complementary treatment strategies for this patient population (147).

The KEYNOTE-495/KeyImPaCT trial, a phase 2 study (NCT03516981), investigated the clinical efficacy of first-line pembrolizumab-based combination therapies in advanced NSCLC using the prospective T cell-inflamed gene expression profile (Tcell_inf_ GEP) and TMB dual biomarker status (148). The patients with previously untreated stage IV NSCLC were categorized into different biomarker-defined subgroups and assigned to receive pembrolizumab in combination with lenvatinib, quavonlimab, or favezelimab. Adaptive randomization based on ORR and frequent interim analyses were employed, with the primary endpoint being investigator-assessed ORR per Response Evaluation Criteria in Solid Tumors (RECIST) v1.1. At the data cutoff in March 2022, the study included 243 patients, and the efficacy data demonstrated that pembrolizumab plus lenvatinib met the prespecified efficacy threshold in the Tcell_inf_ GEP^non-low^ TMB^non-high^ subgroup. Pembrolizumab-based combination therapy exhibited promising antitumor activity and durable response, particularly in the Tcell_inf_ GEP^non-low^ TMB^high^ subgroup across all combinations. While the pembrolizumab plus favezelimab arm did not reach the efficacy bar, there was a notable trend toward improved ORR in the Tcell_inf_ GEP^non-low^ TMB^high^ subgroup. Median PFS and OS were also numerically longer in this subgroup than others. The study suggests that prospectively assessing dual biomarkers can aid in identifying NSCLC patients most likely to respond to pembrolizumab-based combination therapies (148).

There is increasing evidence that proton pump inhibitors (PPIs) influence the growth and survival of cancers; they are one of the most widely used drugs worldwide (149). A study on the impact of PPIs on cancer treatment outcomes, specifically with ICIs, focused on participants with chemotherapy-naïve mNSCLC who were randomized into different treatment arms involving atezolizumab plus carboplatin plus paclitaxel (ACP), bevacizumab plus carboplatin plus paclitaxel (BCP), or atezolizumab plus BCP (ABCP) (150). The analysis revealed that 37% of the 1,202 participants received a PPI, and PPI use was independently associated with worse OS and PFS in the pooled atezolizumab arms (ACP plus ABCP). This negative association was not observed in the BCP arm. Notably, the detrimental effect of PPI use on OS was more pronounced in patients receiving atezolizumab compared to those receiving bevacizumab. The findings propose that PPIs may negatively impact the effectiveness of ICIs, underlining the need for further exploration of the interplay between PPIs and immunotherapy outcomes in cancer treatment (150). It has been revealed that PPIs can modulate the efficacy of antineoplastic agents, such as oral and intravenous chemotherapy, TKIs, and ICIs, by interacting with the cancer microbiome. However, it is notable that due to the limited number of patients participating in retrospective cohort studies, data on drug–drug interactions are limited, and further pharmacoepidemiological studies are needed. Considering the pathophysiology of PPI administration in the context of cancer-related treatment, significant drug–drug interactions, dysbiosis, and appropriate prescribing should be regarded (149).

To summarize this section, diverse therapeutic approaches are emerging as potential advances in managing NSCLC, as demonstrated by the reviewed studies. In locally advanced unresectable NSCLC, immunotherapy, particularly PD-L1 inhibitors like durvalumab, has shown remarkable efficacy as consolidation therapy after chemotherapy and radiotherapy, demonstrating prolonged OS by suppressing inhibitory molecules in the TME and inducing antitumor immune responses (151). Ongoing investigations, like the IMpower010 and Keynote 091 trials, explored the adjuvant setting, revealing promising DFS advantages with agents like atezolizumab and pembrolizumab, particularly in cases with high PD-L1 expression. Notably, a retrospective analysis explores the efficacy of ICIs in NSCLC with *KRAS* mutations, advocating for chemo-ICI combinations as first-line strategies. The combination of camrelizumab and low-dose apatinib demonstrates encouraging outcomes in advanced non-squamous NSCLC, irrespective of PD-L1 expression.

Furthermore, novel strategies involving IL-6 inhibition in *EGFR*-mutant NSCLC with acquired EGFR-TKI resistance point to potential synergic effects between targeted therapies and immunomodulation. Additionally, real-world evidence highlights the favorable impacts of first-line pembrolizumab-based combinations in metastatic NSCLC, emphasizing the importance of these approaches beyond clinical trial settings. These insights collectively support the notion that integrating immunotherapeutic strategies, exploring combinatorial approaches, and addressing specific molecular subtypes hold promise for significantly improving outcomes in NSCLC patients. The evolving landscape of immunotherapy and targeted interventions provides a diversified armamentarium, offering hope for enhanced and personalized therapeutic avenues in the challenging domain of advanced NSCLC. All data presented in this subsection are shown in **Table 1**.

**Table 1:** The most important combination therapies using ICIs for treating NSCLC.

Intervention	Type of NSCLC and study	Outcome	Ref/NCT
**Pembrolizumab + chemotherapy**	Patients with NSCLC and MPE undergoing ICI therapy	○ **PD-L1 high cohort (143 patients):** ○ After PSM, ICI/Chemo showed a significantly prolonged median PFS compared to pembrolizumab monotherapy. ○ Median PFS: 11.1 months (ICI/Chemo) versus 3.9 months (pembrolizumab monotherapy; p = 0.0409). ○ **ICI/Chemo cohort (139 patients):** ○ Some regimens featured bevacizumab (BEV) known for MPE control. ○ 23 patients received BEV. ○ PSM analysis revealed no significant difference in median PFS between the BEV and non-BEV groups. ○ Median PFS: 6.1 months (BEV) versus 7.4 months (non-BEV; p = 0.9610).	(116)
**Platinum + pemetrexed + pembrolizumab**		○ **Association with PFS:** ○ Better performance status (0–1) linked to improved PFS. ○ Higher PD-L1 tumor proportion score (≥50%) associated with enhanced PFS. ○ **Real-world efficacy:** ○ Combination treatment less effective in patients with poor performance status. ○ **Age and performance status impact on adverse events:** ○ Severe adverse events more common in older individuals. ○ Individuals with poor performance status experienced a higher occurrence of severe adverse events compared to healthier counterparts. ○ **Treatment discontinuation:** ○ Substantial number of patients experienced adverse events leading to treatment discontinuation. ○ Higher rate of treatment discontinuation in older individuals.	(117)
**Pembrolizumab + pemetrexed + carboplatin**	Patients with metastatic NSCLC without specific genetic alterations (*EGFR/ALK/ROS1*)	○ **OS:** ○ Median OS: 17.2 months. ○ **Treatment duration:** ○ Median treatment duration for pembrolizumab: 5.8 months. ○ **Real-world response rate:** ○ Response rate: 39.3%. ○ **Survival rates at different time points:** ○ 12-month survival rate. ○ 24-month survival rate. ○ Variability observed across different PD-L1 expression levels. ○ **PD-L1 expression and outcomes:** ○ Higher PD-L1 expression associated with somewhat better outcomes. ○ **Real-world PFS:** ○ Median PFS: 6.2 months. ○ **Duration of response:** ○ Median duration of response: 13.1 months.	(118)
**Pembrolizumab + chemotherapy**	Patients with metastatic NSCLC who developed resistance to EGFR-TKIs	○ **Comparison with classical chemotherapy:** ○ ICI-based therapy significantly improved OS. ○ HR: 0.55 (95% CI, 0.34–0.88; p = 0.01). ○ **Combination with chemotherapy:** ○ The combination of ICI and chemotherapy demonstrated sustained OS benefit. ○ **Subgroup analysis:** ○ Sustained OS benefit observed across various subgroups, including: ○ Younger patients (<65 years). ○ Those without secondary T790M mutations. ○ Individuals without liver and brain metastases. ○ Particularly notable in those with good ECOG scores.	(119)
**Pembrolizumab + chemotherapy**	Patients with NSCLC harboring *EGFR* mutations who have experienced resistance to EGFR TKIs	○ **Median PFS in ICI-treated NSCLC patients:** ○ Overall median PFS: 5.6 months. ○ **Stratification by treatment strategy:** ○ ICI monotherapy group: median PFS of 2.4 months. ○ ICI combined with chemotherapy group: median PFS of 5.9 months. ○ **Broader analysis across multiple studies:** ○ Included 16 studies (5 trials, 10 controlled cohorts, and 1 real-world study) for ICI-treated NSCLC patients with EGFR mutations after TKI failure. ○ 6-month survival rate: 0.82 (95% CI, 0.36–0.97). ○ PFS rate: 0.55 (95% CI, 0.34–0.74). ○ **Best survival outcome with combination therapy:** ○ ICI combined with chemotherapy demonstrated the best survival outcome. ○ Indicated by the 12-month survival rate and PFS. ○ **Safety profile:** ○ No new safety signals identified with combination therapy. ○ Frequency of treatment-related adverse events similar to previous studies of chemotherapy combined with checkpoint inhibitors.	(120)
**Camrelizumab + chemotherapy**	Patients with stage IIIB-IV squamous NSCLC A double-blind, randomized, phase 3 trial	○ **PFS:** ○ Combination of camrelizumab and chemotherapy significantly extended PFS. ○ Median PFS: 8.5 months (combination) vs. 4.9 months (placebo-chemotherapy). ○ p < 0.0001. ○ **OS:** ○ Combination of camrelizumab and chemotherapy significantly extended OS. ○ Median OS: Not reached (combination) vs. 14.5 months (placebo-chemotherapy). ○ p < 0.0001. ○ **Adverse events:** ○ No unexpected irAEs observed in either treatment group. ○ **Biomarker analysis [circulating tumor DNA (ctDNA)]:** ○ Focused on ctDNA dynamics. ○ ctDNA clearance after two cycles of treatment independently associated with considerably longer PFS and OS. ○ p < 0.0001 for both.	(121) NCT03668496
**Pembrolizumab + chemotherapy**	KEYNOTE-789 study, a phase III randomized, double-blind trial in patients with TKI-resistant, *EGFR*-mutant, metastatic non-squamous NSCLC	○ **Interim analysis (IA2):** ○ At IA2, median PFS was 5.6 months with pembro + chemo versus 5.5 months with pbo + chemo, without statistical significance. ○ **Final analysis (FA):** ○ At FA (data cutoff: January 17, 2023), median OS was 15.9 months with pembro + chemo versus 14.7 months with pbo + chemo. HR favored pembro + chemo (0.84) but didn’t reach significance. ○ **Response rates and duration of response:** ○ ORR and DOR were similar between the two groups. ○ **Adverse events:** ○ Grade ≥3 treatment-related AEs occurred in 43.7% of pembro + chemo patients and 38.6% of pbo + chemo patients. ○ Immune-mediated AEs and infusion reactions were higher in the pembro + chemo arm but generally manageable. ○ No new safety signals were identified.	(122)
**Pembrolizumab + SBRT**	Advanced-stage NSCLC PEMBRO-RT trial	○ **Lymphocyte changes:** ○ Significant increase in the total number of lymphocytes observed after 6 weeks of treatment. ○ Observed in both anti-PD-1 alone and anti-PD-1 + SBRT groups. ○ **Impact of SBRT and anti-PD-1 combination:** ○ Combination of SBRT and anti-PD-1 resulted in a substantial rise in CD103^+^ cytotoxic T cells. ○ **Baseline comparison for responders and non-responders:** ○ Responders exhibited a higher baseline lymphocyte count compared to non-responders.	(123)
**Ipilimumab + paclitaxel + carboplatin**	A randomized, double-blind, phase III study chemotherapy-naïve patients with stage IV or recurrent squamous NSCLC	○ **Findings:** ○ Combination therapy did not significantly prolong OS compared to chemotherapy alone. ○ **Median OS:** ○ 13.4 months for chemotherapy plus ipilimumab and 12.4 months for chemotherapy plus placebo. ○ **PFS:** ○ Was similar in both groups. ○ **Safety profile:** ○ Higher rates of grade 3 or 4 treatment-related AEs with chemotherapy plus ipilimumab compared to chemotherapy plus placebo. ○ Chemotherapy plus ipilimumab associated with seven treatment-related deaths, while chemotherapy plus placebo had one.	(125)
**Nivolumab + ipilimumab**	First-line treatment in patients with advanced NSCLC	○ **Median follow-up:** ○ 54.8 months ○ **OS:** ○ Remained significantly longer with nivolumab plus ipilimumab compared to chemotherapy in both PD-L1 ≥1% and <1% subgroups. ○ **HR:** ○ 0.76 (PD-L1 ≥ 1%) and 0.64 (PD-L1 < 1%), indicating a significant decrease in the risk of death. ○ **4-year OS rates:** ○ 29% versus 18% (PD-L1 ≥ 1%) and 24% versus 10% (PD-L1 < 1%) for nivolumab plus ipilimumab versus chemotherapy, respectively. ○ **Safety profile:** ○ Consistent with previous studies. ○ Rash was the most common irAE. ○ Early occurrence of irAEs managed according to guidelines.	(126)
**Durvalumab + tremelimumab**	Patients with advanced NSCLC	○ **Sub-study A findings:** ○ Significant advancements in treatment outcomes for heavily pretreated mNSCLC patients with durvalumab compared to standard of care (SoC). ○ **Median OS with durvalumab:** ○ 11.7 months, surpassing 6.8 months with SoC ○ **HR for mortality:** ○ 0.63 ○ **Improvement in PFS with durvalumab:** ○ Median duration 3.8 months compared to 2.2 months with SoC. ○ **HR:** ○ 0.71 ○ **Treatment-related grade 3/4 AEs:** ○ 9.7% with durvalumab versus 44.4% with SoC, highlighting a notable disparity.	(127) NCT02352948
**Durvalumab + tremelimumab**	Non-randomized, open-label, phase 1b trial in patients with advanced squamous or NSCLC	○ **Adverse events:** ○ Most common treatment-related grade 3 and 4 AEs: diarrhea, colitis, and increased lipase. ○ **Discontinuations due to treatment-related AEs:** ○ 28% of patients ○ **Serious treatment-related AEs:** ○ 36% of patients ○ Twenty-two deaths during the study, with three attributed to treatment-related complications (myasthenia gravis, pericardial effusion, and neuromuscular disorder). ○ **Clinical activity:** ○ Despite adverse events, clinical activity observed in patients with PD-L1^+^ and PD-L1^−^ tumors. ○ **ORR:** ○ Achieved by 23% of patients in combined tremelimumab 1 mg/kg cohort, with responses in both PD-L1^+^ and PD-L1^−^ tumors.	(128)
**Vibostolimab + pembrolizumab**	Phase 1 study on anti-PD-1/PD-L1-naïve NSCLC patients, with 73% having received at least one prior line of therapy	○ Well-tolerated regimen in patients with advanced solid tumors during dose- escalation/confirmation phase. ○ **Common treatment-related AEs:** ○ Pruritus (34%), hypoalbuminemia (29%), pyrexia (20%). ○ Grade 3–4 treatment- related AEs: occurred in 15% of patients, with no treatment-related AE deaths reported. ○ **Clinical response:** ○ Median duration of response not reached, ranging from 4 to 17+ months	(129) NCT02964013
**Sabatolimab + spartalizumab**	Phase I/II clinical trial	○ **Maximum tolerated dose (MTD) and adverse events:** ○ MTD not reached. ○ Fatigue was the most common treatment-related AE. ○ **Clinical response:** ○ Sabatolimab monotherapy showed no responses. ○ Combination with spartalizumab resulted in partial responses in colorectal cancer, NSCLC, malignant perianal melanoma, and SCLC. ○ **Duration of responses:** ○ 12 to 27 months ○ **Biomarker analysis:** ○ Two responsive patients had elevated expression of immune markers in baseline biopsies. ○ Three patients had more than 10% TIM-3+ staining, including one with NSCLC who had prior PD-1 therapy. ○ Promising tolerability and preliminary signs of antitumor activity observed	(130)
**Anti-PD-1 + Ad5-CMV-mTNFα/mIL-2**	Mouse NSCLC model	○ **Combined treatment efficacy:** ○ Notably reduced cancer growth in animals. ○ Effective even in the presence of neutralizing antibodies. ○ **Mechanisms of action:** ○ Increased cytotoxic tumor-infiltrating lymphocytes, especially tumor-specific cells. ○ Decreased immunosuppressive tumor-associated macrophages. ○ Improved maturation of DCs. ○ **Tumor-specific memory T cells:** ○ Group receiving anti-PD-1 with armed virus showed expansion in the tumor- specific memory T-cell compartment within secondary lymphoid organs. ○ **Limitations and addressing concerns:** ○ Non-replicative nature of Ad5-CMV-mTNFα/mIL-2 virus in the murine model. ○ Concerns about its complete reflection of human clinical outcomes. ○ Researchers complemented findings using NSCLC *ex vivo* models. ○ ***Ex vivo* model confirmation:** ○ NSCLC *ex vivo* models fully permitted TNF-α and IL-2-armed oncolytic adenovirus TILT-123 activity.	(131)
**Depleting IL-6 + anti-PD-1**	*EGFR-*mutant genetically engineered mouse models	○ ***EGFR*-mutant tumors with acquired resistance:** ○ Displayed a mesenchymal phenotype. ○ Showed significantly increased IL-6 secretion. ○ **In *EGFR*-mutant GEMMs:** ○ Depleting IL-6: ○ Enhanced activation of infiltrating natural killer (NK) and T-cell subpopulations. ○ Reduced immunosuppressive regulatory T and Th17 cell populations. ○ **IL-6 inhibition in cell culture:** ○ Increased NK- and T cell-mediated killing of human osimertinib-resistant EGFR- mutant NSCLC tumor cells. ○ **IL-6 blockade sensitization:** ○ Sensitized EGFR-mutant GEMM tumors to PD-1 inhibitors. ○ Increased tumor-infiltrating IFNγ^+^ CD8^+^ T cells.	(136)
**Erlotinib + anti-PD-1**	Human samples *In vitro*/*in vivo*	○ Erlotinib monotherapy reduces the infiltration of CD4^+^ effector Tregs in the TME. ○ Combining erlotinib with anti-PD-1 could be more effective than monotherapies. ○	(141)
**Anti-PD-L1 + anti-CD73 antibodies**	Xenograft mouse model representative of *EGFR*-mutated NSCLC	○ **Single antibody administration in EGFR-mutated NSCLC model:** ○ Individual anti-PD-L1 or anti-CD73 antibody administration: ineffective in curtailing tumor growth. ○ Isotype control had a different outcome. ○ **Combined antibody administration:** ○ Combined administration of anti-PD-L1 and anti-CD73 antibodies: significantly retards tumor growth. ○ Notable contrast to individual antibody treatments. ○ **Effect on tumor-infiltrating CD8^+^ T cells:** ○ Combined treatment amplifies the presence of tumor-infiltrating CD8^+^ T cells. ○ **Cytokine production by CD8^+^ T cells:** ○ Combined treatment enhances the production of IFN-γ and TNF-α by tumor- infiltrating CD8^+^ T cells. ○ **Gene expression changes:** ○ Parallel elevation in gene expression associated with inflammation. ○ Enhanced T-cell function observed in tumors subjected to the combination therapy.	(144)
**Camrelizumab + apatinib**	Absence of *EGFR* or *ALK* alterations	○ **ORR:** 40.0% ○ The majority of patients achieved either partial responses or stable disease. ○ **Disease control rate:** 92.0% ○ Reflecting a high level of control over disease progression. ○ **PFS:** 9.6 months ○ Indicates a significant delay in disease advancement. ○ **OS:** ○ Median OS was not reached at the time of reporting. ○ Suggests a potential for prolonged survival benefits. ○ **PD-L1 expression:** ○ Positive clinical activity was consistent across patients regardless of PD-L1 expression levels. ○ **Safety profile:** ○ Generally acceptable. ○ **Common grade 3 or higher adverse events:** ○ Increased gamma-glutamyl transferase. ○ Increased alanine aminotransferase. ○ Abnormal hepatic function.	(145)
**Teliso-V (telisotuzumab vedotin) + nivolumab**	Advanced NSCLC patients Phase 1b study	○ **Common treatment-related adverse events (any grade):** ○ Fatigue: 27% ○ Peripheral sensory neuropathy: 19% ○ **Pharmacokinetic profile:** ○ Teliso-V plus nivolumab comparable to Teliso-V monotherapy. ○ **Antitumor activity:** ○ Limited overall, with an objective response rate of 7.4%. ○ **Confirmed partial responses:** ○ Two patients with PD-L1 positivity and c-Met immunohistochemistry H-score of 190. ○ One patient with PD-L1 negativity and c-Met H-score of 290. ○ **PFS:** ○ Overall median: 7.2 months. ○ Variation among different PD-L1 subgroups (PD-L1+: 7.2 months; PD-L1–: 4.5 months; PD-L1-unknown: not reached).	(147) NCT02099058
**Pembrolizumab + lenvatinib + quavonlimab, or favezelimab**	Stage IV NSCLC KEYNOTE-495/KeyImPaCT trial, a phase 2 study	○ **Common treatment-related adverse events (any grade):** ○ Fatigue: 27% ○ Peripheral sensory neuropathy: 19% ○ **Pharmacokinetic Profile:** ○ Teliso-V plus nivolumab comparable to Teliso-V monotherapy. ○ **Antitumor activity:** ○ Limited overall, with an objective response rate of 7.4%. ○ **Confirmed partial responses:** ○ Two patients with PD-L1 positivity and c-Met immunohistochemistry H-score of 190. ○ One patient with PD-L1 negativity and c-Met H-score of 290. ○ **PFS:** ○ Overall median: 7.2 months. ○ Variation among different PD-L1 subgroups (PD-L1+: 7.2 months; PD-L1–: 4.5 months; PD-L1-unknown: not reached).	(148) NCT03516981
**Atezolizumab plus carboplatin plus paclitaxel (ACP), bevacizumab plus carboplatin + paclitaxel (BCP), or atezolizumab + BCP (ABCP)**	Chemotherapy-naïve, metastatic NSCLC	○ **PPI use:** ○ 37% of participants received a proton pump inhibitor (PPI). ○ **Association with outcomes in atezolizumab arms (ACP plus ABCP):** ○ PPI use independently associated with worse OS and PFS. ○ **Comparison with bevacizumab arm (BCP):** ○ Negative association of PPI use not observed in the BCP arm. ○ **Differential impact of PPI use:** ○ Detrimental effect of PPI use on OS more pronounced in patients receiving atezolizumab compared to those receiving bevacizumab.	(150)

ICIs, immune checkpoint inhibitors; NSCLC, non-small cell lung cancer; MPE, malignant pleural effusion; PD-L1, programmed death-ligand 1; PSM, propensity score matching; PFS, progression-free survival; OS, overall survival; EGFR, epidermal growth factor receptor; TKIs, tyrosine kinase inhibitors; ECOG, Eastern Cooperative Oncology Group; irAEs, immune-related adverse events; ctDNA, circulating tumor DNA; pembro + chemo, pembrolizumab with chemotherapy; pbo + chemo, placebo with chemotherapy; ORR, objective response rate; DOR, duration of response; SBRT, stereotactic body radiation therapy; mNSCLC, metastatic NSCLC; AEs, adverse events; DCs, dendritic cells; GEMMs, genetically engineered mouse models; Tregs, regulatory T cells; TME, tumor microenvironment.

### 5.4 Targeted agents

According to the available knowledge, *ALK* rearrangements in NSCLC prompt using ALK TKIs such as crizotinib, ceritinib, and alectinib, with sequential treatment often required to address resistance mechanisms (152). Similarly, *ROS1* rearrangements find targeted therapy in crizotinib (153). Vascular endothelial growth factor receptor (VEGFR) TKIs, including sorafenib and sunitinib, target angiogenesis and are part of the therapeutic arsenal (154). HER2 TKIs, afatinib, and neratinib hold promise for NSCLC patients with *HER2* mutations or amplifications (155). On May 28, 2021, the FDA granted accelerated approval to sotorasib (Lumakras, Amgen) for treating adults with advanced NSCLC carrying a *KRAS G12C* mutation who have undergone at least one prior systemic therapy (156). This milestone marked the first approval of a targeted therapy for *KRAS G12C*-mutated NSCLC. The approval decision was informed by the results of the CodeBreaK 100 trial (NCT 20170543), which encompassed both dose-escalation and dose-expansion phases involving patients with advanced, *KRAS G12C*-mutated, solid tumors (157). Among patients with *KRAS G12C*-mutated NSCLC treated with sotorasib (n = 124), the ORR was 36%, with a median duration of response of 10.0 months. These findings provided a basis for the accelerated approval, highlighting the potential efficacy of sotorasib in this specific patient population. Remarkable adverse reactions (≥20%) associated with sotorasib treatment included diarrhea, musculoskeletal pain, nausea, fatigue, hepatotoxicity, and cough. The safety profile informs clinicians about the commonly observed side effects. Due to pharmacokinetic data and ORRs observed in patient cohorts administered lower doses during the dose-escalation phase of CodeBreaK 100, a dose comparison study is underway as a post-marketing requirement. This initiative aims to refine the optimal dosage of sotorasib for patients with *KRAS G12C*-mutated NSCLC (157). The FDA’s accelerated approval of sotorasib represents a significant advancement in the treatment landscape for patients with *KRAS G12C*-mutated NSCLC who have exhausted prior systemic therapies. The ongoing dose comparison study emphasizes the commitment to optimizing treatment strategies and furthering our understanding of this targeted therapy.

Osimertinib, another third-generation EGFR TKI, is the frontline standard for treating metastatic *EGFR*-mutant NSCLC (158). Despite its efficacy, nearly universal disease progression ensues in patients, propelled by a heterogeneous spectrum of resistance mechanisms (159). While *MET* signaling pathway activation through amplification has been recognized as a factor in osimertinib resistance, the involvement of point mutations in *MET* activation still needs to be more adequately characterized (160). A case study reported a 65-year-old woman with metastatic *EGFR*-mutant NSCLC manifesting disease progression on osimertinib due to the emergence of a *MET Y1003N* mutation (161). Subsequent administration of capmatinib in combination with osimertinib produced a partial response. This case illuminates the potential of dual EGFR/MET inhibition in instances of *EGFR*-mutated NSCLC, mainly when activation of *MET* mutations instigates resistance (161). These findings contribute to a nuanced understanding of resistance mechanisms and offer a promising therapeutic avenue for consideration within this specific patient subset.

An investigation explored the therapeutic potential of a novel combination involving trastuzumab emtansine (T-DM1) and osimertinib for patients with mNSCLC characterized by the presence of *EGFR* mutations (*EGFR*m^+^) and resistance to osimertinib treatment (162). Although EGFR TKIs have shown substantial success in improving the survival of such patients, the emergence of resistance, often accompanied by HER2 protein overexpression, poses a significant clinical challenge. The central hypothesis posits that concurrent inhibition of EGFR and HER2 using osimertinib and T-DM1 could reinstate tumor responsiveness. The study adopts a multicenter, single-arm, phase 1–2 design (NCT03784599), encompassing patients with *EGFR*m^+^ NSCLC displaying HER2 overexpression while experiencing progression on osimertinib. The treatment protocol involves intravenous administration of T-DM1 at a dose of 3.6 mg/kg every 3 weeks and daily oral osimertinib at 80 mg. Primary endpoints encompass the ORR at the 12-week juncture and safety assessments. Methodological rigidity is maintained by applying Simon’s two-stage minimax design, with predetermined thresholds for ORR, statistical power, and type I error rates. Spanning the recruitment period from January 2019 to April 2021 and encompassing 27 enrolled patients, the trial yielded an objective response rate of 4% after 12 weeks of treatment. Median PFS was reported at 2.8 months, with fatigue, diarrhea, and nausea emerging as the most prevalent treatment-related AEs, predominantly of grade 3 severity. Notably, no grade 4 or 5 therapy-related AEs were recorded. Taken together, the TRAEMOS trial (T-DM1 and osimertinib) represents a pioneering effort in investigating the synergistic potential of T-DM1 and osimertinib for patients with *EGFR*m^+^ NSCLC exhibiting HER2 overexpression upon osimertinib resistance. Despite a favorable safety profile compared to conventional cytotoxic chemotherapy, the treatment regimen demonstrates limited efficacy, as evidenced by the low objective response rate and a short PFS period. Based on these findings, the authors advocate against further clinical exploration of this combination (162).

The combination of alectinib and atezolizumab as a first-line treatment for advanced *ALK*-positive NSCLC, focusing on assessing safety, tolerability, and potential antitumor activity, was evaluated by a study (163). The two-stage, phase 1b trial enrolled treatment-naïve adults with stage IIIB/IV or recurrent *ALK*-positive NSCLC. The patients received alectinib 600 mg twice daily throughout each 21-day cycle, along with atezolizumab 1,200 mg on day 8 of cycle 1 and then on day 1 of each subsequent 21-day cycle. The primary objectives were to evaluate the safety and tolerability of the combination, while secondary objectives included assessments of antitumor activity. No dose-limiting toxicities were observed in the first stage of the study, involving seven patients. This led to the continuation of the starting dose and schedule into the second stage, involving 14 additional patients. The median duration of follow-up was 29 months, ranging from 1 to 39 months. The results indicated that 57% of the patients experienced grade 3 treatment-related AEs, with rash being the most common (19%). However, no grade 4 or 5 treatment-related AEs were reported. The confirmed objective response rate was 86% (18 out of 21 patients), with a 95% confidence interval of 64%–97%. The median PFS and OS were not estimable within the given confidence intervals. While the combination of alectinib and atezolizumab was feasible, the study noted increased toxicity compared to the individual agents. It is essential to highlight that the small sample size and relatively short follow-up limit definitive conclusions regarding antitumor activity (163). These data provide valuable insights into the safety and initial efficacy of the combination therapy in the specified patient population. However, further research with larger sample sizes and extended follow-up periods is warranted to draw more definitive conclusions about the antitumor activity and overall efficacy of the alectinib and atezolizumab combination for *ALK*-positive NSCLC.

A study focused on the approval and efficacy of amivantamab for treating advanced NSCLC characterized by *EGFR* exon 20 insertions, particularly in patients experiencing disease progression during or after platinum-based chemotherapy (164). Phase 1 data have previously demonstrated the safety and antitumor activity of amivantamab, especially when combined with carboplatin-pemetrexed chemotherapy. However, further investigation through an international, randomized, phase 3 trial was conducted to provide additional insights into this combination therapy. In this phase 3 trial (NCT04538664), patients with advanced NSCLC and *EGFR* exon 20 insertions who had not undergone previous systemic treatment were randomly assigned in a 1:1 ratio to receive either intravenous amivantamab plus chemotherapy (amivantamab–chemotherapy) or chemotherapy alone (164). The primary outcome measured was PFS, evaluated through an anonymous independent central review. Notably, patients in the chemotherapy-only group experiencing disease progression were allowed to cross over to receive amivantamab monotherapy. The results from the study encompassing 308 randomized patients (153 in the amivantamab + chemotherapy group and 155 in the chemotherapy-alone group) revealed a significantly longer PFS in the amivantamab + chemotherapy group compared to the chemotherapy-alone group, and the median PFS was 11.4 months and 6.7 months, respectively, with a hazard ratio for disease progression or death 0.40 (95% CI, 0.30 to 0.53). The primary AEs associated with amivantamab + chemotherapy were reversible hematologic effects and EGFR-related toxic effects. Notably, 7% of patients discontinued amivantamab due to adverse reactions (164). The findings indicate that the use of amivantamab + chemotherapy demonstrates superior efficacy compared to chemotherapy alone as a first-line treatment for patients with advanced NSCLC exhibiting *EGFR* exon 20 insertions. The observed longer PFS and manageable AEs support the potential of amivantamab as a promising therapeutic option in this specific patient population.

In a distinct subgroup of NSCLC, comprising approximately 1%–3%, *HER2* mutations are recognized as genomic drivers, yet no HER2-targeted treatment has gained official approval for NSCLC. Within the Drug Rediscovery Protocol (DRUP) framework, a study sought to evaluate the efficacy of a combination therapy involving trastuzumab and pertuzumab in patients with NSCLC harboring *HER2* exon 20 mutations (165). The trial enrolled patients with advanced *HER2*-mutated NSCLC that was refractory to treatment, with the primary endpoint being clinical benefit (CB), defined as either achieving an objective response or maintaining stable disease for a minimum of 16 weeks. Utilizing a Simon-like 2-stage design, 24 evaluable patients were enrolled. The findings revealed CB in 38% of patients, including an objective response rate of 8.3% and seven patients exhibiting stable disease for at least 16 weeks. The predominant *HER2* mutation identified was p.Y772_A775dup (71%). Despite meeting the primary endpoint, the trastuzumab/pertuzumab combination demonstrated only marginal efficacy in a subset of heavily pretreated patients with *HER2*-mutated NSCLC. Median PFS stood at 4 months and OS at 10 months, indicating a modest clinical impact. Whole-genome sequencing validated the inclusion mutation in all cases; no unexpected toxicities were noted (165). Therefore, while the combination therapy yielded some clinical benefit, its overall activity was limited in this specific subgroup of patients with *HER2*-mutated NSCLC undergoing extensive prior treatments.

In a two-stage, phase 2 study, the researchers aimed to evaluate the treatment efficacy of combining bevacizumab with atezolizumab in patients with mNSCLC whose disease had progressed following atezolizumab monotherapy (166). The investigation was grounded in the understanding that vascular endothelial growth factor (VEGF) contributes to an immunosuppressive tumor microenvironment, which antiangiogenic therapies like bevacizumab can counteract. ICI-naïve NSCLC patients without specific genetic alterations were enrolled. Initial treatment with atezolizumab yielded a disease control rate (DCR) of 35.7% (stage I). Subsequent addition of bevacizumab to atezolizumab in stage II resulted in an impressive DCR of 87.5%, with notable partial responses and stable disease. Median PFS and OS in stage II were 5.6 and 14.0 months, respectively. Importantly, treatment-related AEs in stage II were mild (grade 1 or 2) in 25% of patients. These findings highlight the potential of combining bevacizumab with atezolizumab as a promising and well-tolerated therapeutic strategy for mNSCLC patients progressing after atezolizumab monotherapy, offering valuable insights into future treatment approaches (166).

The POLISH study addressed a notable gap in understanding first-line treatment strategies for advanced NSCLC with *HER2* alterations, a subject that has yet to be explored in large-scale studies. Conducted retrospectively between November 2015 and September 2021, the study screened 293 patients with HER2-altered NSCLC, identifying HER2 amplification and 37 distinct *HER2* mutations. Of the total, 210 patients who received first-line chemotherapy alone (C), chemotherapy plus ICIs (C + I), or chemotherapy plus angiogenesis inhibitors (C + A) were included in the final efficacy analysis. The study revealed that the combination of chemotherapy and angiogenesis inhibitors (C + A) conferred a significant improvement in PFS compared to chemotherapy alone (C), with a median PFS of 5.63 months versus 4.03 months, supported by an HR of 0.64 and a confidence interval of 0.46–0.88 (p = 0.006). Conversely, the addition of ICIs (C + I) did not demonstrate a PFS advantage compared to C + A or C, despite considerations such as PD-L1 expression or tumor mutational burden. Noteworthy molecular insights emerged from the Kyoto Encyclopedia of Genes and Genomes (KEGG) analysis, indicating a common upregulation of the PI3K/AKT pathway signaling in *HER2*-altered NSCLC. In conclusion, the study suggests that combining chemotherapy with angiogenesis inhibitors may offer a superior survival benefit in the first-line setting for *HER2*-altered NSCLC while indicating that immune-based combination therapy may not surpass the efficacy of chemotherapy alone. The identified upregulation of the PI3K/AKT pathway signaling could mediate immunosuppression in this context (167).

A case study involving a patient with *ALK* ^+^ NSCLC, a condition often initially responsive to TKIs but prone to eventual drug resistance, reported that the patient, who had undergone multiple lines of TKI treatment, disclosed promising therapeutic outcomes when treated with a combination of SBRT and pneumococcal conjugate vaccine (PCV) (168). The patient’s metastatic lesion, located in the lumbar spine, exhibited regression following SBRT. Surprisingly, the patient also experienced an abscopal complete pathological response (CPR) after the combined use of SBRT and PCV. Biopsy analysis of the primary lung lesion revealed map-like necrosis and infiltration by TILs. Moreover, multifocal granulomas and early tertiary TLS were observed. This case suggests combining radiotherapy with PCV may elicit a specific immune response and reshape the TME in TKI-resistant NSCLC. The formation of granulomas and TLS, along with the infiltration of TILs, indicates a potential immune-stimulating effect. These findings provide a novel perspective for future immunotherapy approaches in the challenging clinical scenario of TKI-resistant NSCLC. As demonstrated in this case, combining SBRT with immunotherapy may offer a promising strategy for enhancing treatment responses in patients with advanced ALK^+^ NSCLC (168). **Table 2** displays all pertinent data.

**Table 2:** The most important combination therapies using TKIs for treating NSCLC.

Intervention	Type of NSCLC and study	Outcome	Ref/NCT
**Trastuzumab emtansine (T-DM1) + osimertinib**	Patients with metastatic NSCLC characterized by the presence of *EGFR* mutations (*EGFR*m^+^) and resistance to osimertinib treatment A multicenter, single-arm, phase 1–2 design	○ **Median PFS:** ○ Reported at 2.8 months. ○ **Prevalent treatment-related adverse events:** ○ Fatigue, diarrhea, and nausea emerged as the most prevalent treatment-related adverse events. ○ **Severity of adverse events:** ○ Predominantly of grade 3 severity. ○ **Severe adverse events:** ○ No grade 4 or 5 therapy-related adverse events were recorded.	(162) NCT03784599
**Alectinib + atezolizumab**	The two-stage, phase 1b trial enrolled treatment-naïve adults with stage IIIB/IV or recurrent *ALK*-positive NSCLC	○ **Dose-limiting toxicities in the first stage:** ○ No dose-limiting toxicities were observed in the first stage of the study, involving seven patients. ○ **Continuation into the second stage:** ○ The absence of dose-limiting toxicities led to the continuation of the starting dose and schedule into the second stage, involving 14 additional patients. ○ **Median duration of follow-up:** ○ The median duration of follow-up was 29 months, ranging from 1 to 39 months. ○ **Treatment-related adverse events:** ○ 57% of patients experienced grade 3 treatment-related adverse events. ○ Rash was the most common adverse event, observed in 19% of patients. ○ No grade 4 or 5 treatment-related adverse events were reported. ○ **Objective response rate:** ○ The confirmed objective response rate was 86% (18 out of 21 patients). ○ 95% confidence interval: 64–97%. ○ **PFS and OS:** ○ Median PFS and OS were not estimable within the given confidence intervals. ○ **Feasibility and toxicity:** ○ While the combination of alectinib and atezolizumab was feasible, the study noted increased toxicity compared to the individual agents.	(163)
**Amivantamab + chemotherapy**	Advanced NSCLC characterized by *EGFR* exon 20 insertions A phase 3 international, randomized trial	○ **PFS:** ○ The amivantamab + chemotherapy group showed a significantly longer PFS compared to the chemotherapy-alone group. ○ Median PFS: 11.4 months (amivantamab + chemotherapy) vs. 6.7 months (chemotherapy-alone). ○ Hazard ratio for disease progression or death: 0.40 [95% confidence interval (CI), 0.30 to 0.53]. ○ **Adverse events:** ○ Primary adverse events associated with amivantamab + chemotherapy were reversible hematologic effects and EGFR-related toxic effects. ○ **Treatment discontinuation:** ○ Notably, 7% of patients discontinued amivantamab due to adverse reactions.	(164) NCT04538664
**Trastuzumab + pertuzumab**	Patients with NSCLC harboring *HER2* exon 20 mutations	○ **Clinical benefit (CB):** ○ Clinical benefit was observed in 38% of patients. ○ **ORR:** ○ The objective response rate was 8.3%. ○ **Stable disease:** ○ Seven patients exhibited stable disease for at least 16 weeks. ○ **HER2 mutation profile:** ○ The predominant HER2 mutation identified was p.Y772_A775dup (71%). ○ **Primary endpoint:** ○ The trastuzumab/pertuzumab combination met the primary endpoint. ○ **Efficacy in heavily pretreated patients:** ○ Despite meeting the primary endpoint, the combination demonstrated only marginal efficacy in a subset of heavily pretreated patients with HER2-mutated NSCLC. ○ **PFS:** ○ Median PFS was 4 months. ○ **OS:** ○ Median OS was 10 months. ○ **Clinical impact:** ○ The findings indicate a modest clinical impact. ○ **Genomic validation:** ○ Whole-genome sequencing validated the inclusion mutation in all cases. ○ **Toxicities:** ○ No unexpected toxicities were noted.	(165)
**Bevacizumab + atezolizumab**	Patients with metastatic NSCLC whose disease had progressed following atezolizumab monotherapy A phase 2 trial	○ **Stage I treatment:** ○ Initial treatment with atezolizumab yielded a disease control rate (DCR) of 35.7%. ○ **Stage II combination treatment:** ○ Subsequent addition of bevacizumab to atezolizumab in stage II resulted in an impressive DCR of 87.5%. ○ **Responses in stage II:** ○ Notable partial responses and stable disease were observed in stage II. ○ **PFS and OS:** ○ Median PFS in stage II was 5.6 months. ○ Median OS in stage II was 14.0 months. ○ **Safety profile in stage II:** ○ Treatment-related adverse events in stage II were mild (grade 1 or 2) in 25% of patients.	(166)
**First-line chemotherapy alone (C), chemotherapy plus ICIs (C + I), or chemotherapy plus angiogenesis inhibitors (C + A)**	Advanced NSCLC with HER2 alterations POLISH study	○ **(C + A):** ○ The combination of chemotherapy and angiogenesis inhibitors significantly improved PFS compared to chemotherapy alone. ○ Median PFS: 5.63 months (C + A) vs. 4.03 months (C). ○ Hazard ratio: 0.64, confidence interval: 0.46–0.88 (p = 0.006). ○ **(C + I):** ○ The addition of immune checkpoint inhibitors did not demonstrate a PFS advantage compared to either C + A or C. ○ This was observed despite considerations such as PD-L1 expression or tumor mutational burden. ○ **Molecular insights:** ○ KEGG analysis revealed a common upregulation of the PI3K/AKT pathway signaling in HER2-altered NSCLC.	(167)

TKIs, tyrosine kinase inhibitors; NSCLC, non-small cell lung cancer; EGFR, epidermal growth factor receptor; PFS, progression-free survival; OS, overall survival.

Taken together, these targeted therapies, guided by comprehensive molecular profiling, signify a paradigm shift in NSCLC treatment. They offer improved outcomes and foster a more nuanced, individualized approach to patient care. Regular updates to clinical guidelines are crucial for integrating the latest advancements into the evolving landscape of NSCLC therapy.

### 5.5 Combination therapies using cytokines

In cancer immunotherapy, cytokines are deemed essential, mainly when combined with other therapeutic modalities. These could involve ICIs, oncolytic viruses, or their incorporation into vaccines based on DCs or tumor cells (169). The ultimate objective is to capitalize on the positive aspects of cytokines in combating cancer while mitigating their limitations. The aim is to make their application more precise, potent, and safer within comprehensive cancer treatment strategies (170). Previous studies showed a potential synergistic effect between various ILs and ICIs for treating advanced solid tumors. Focusing on specific ILs and their respective modulators, the discussion encompasses their impact on T cells, clinical responses, and overall treatment outcomes (171).

Preclinical studies reported that the *Semliki Forest virus* can encode IL-12 (SFV‐IL12), and this type of virotherapy can induce PD‐L1 expression on tumor cells in an IFNγ‐mediated fashion (172). Furthermore, it has been revealed that IFN‐γ can cause the protein kinase D isoform 2 (PKD2), a main regulator of PD‐L1 expression in human oral squamous carcinoma cells (Chen et al., 2012). Therefore, there is a significant association between cytokines and immune checkpoints because PD-L1 expression can positively affect immunotherapy efficacy. Accordingly, it is possible that combining PD-L1-regulator cytokines with ICIs leads to an enhancement of cancer therapy (173). In addition, the combination of immunostimulatory monoclonal antibodies with local IL-12 can induce tumor regression and increase survival rates. In preclinical studies, IL-12 combined with anti-PD-1 monoclonal antibodies has been shown to eliminate established tumors (172), and for the treatment of breast cancer in an animal model, IL-12-based therapy combined with anti-PD-1 monoclonal antibodies yielded promising results. This investigation utilized cellular vaccines expressing the glycolipid‐anchored form of IL‐12 and immune costimulatory B7‐1 with the PD‐L1 blockade for the treatment (174).

Researchers have discovered resistance mechanisms in murine orthotopic lung tumors treated with systemically delivered IL-12 fused to murine serum albumin (MSA; IL12-MSA). A decreased expression of IL-12R on tumor-reactive CD8^+^ T cells is responsible for this resistance (126). This hurdle was overcome by using IL-2-MSA, which amplified the binding of IL-12-MSA by tumor-reactive CD8^+^ T cells. In mice bearing lung tumors, combining IL-12-MSA with IL-2-MSA synergistically induced CD8^+^ T-cell differentiation, CD4^+^ Treg infiltration, and survival. To minimize the potential dose-limiting toxicity associated with IL-2 and IL-12 combination therapy, the study proposes to deliver engineered analogs of IL-12 and IL-2 preferentially bound to cells expressing Il12rb1 and CD25. It suggests a rational and promising avenue for combinatorial cytokine therapy in cancer immunotherapy, extending the survival and mitigating the toxicity of mice with lung tumors (126).

Another preclinical study explored the combination of N-803 (ALT‐803), an IL-15 superagonist mutant, and an anti-PD-L1 antibody in preclinical models of solid cancers resistant to individual treatments. Unlike prior studies, they administered N-803 and the antibody subcutaneously. The combination demonstrated superior efficacy, reducing 4T1 lung metastasis and MC38-CEA tumor burden while enhancing survival compared to N-803 and anti-PD-L1 monotherapies. N-803 upregulated PD-L1 expression on the surface of immune cells, justifying the combination. The therapy’s success depended on CD8^+^ T and NK cells, with N-803 mainly driving alterations in cell phenotype. The addition of anti-PD-L1 significantly boosted CD8^+^ T-cell effector function, increasing serum levels of IFNγ without related toxicities and improving antitumor efficacy. These outcomes support the clinical potential of N-803 in combination with ICIs for minimally responsive patients (175).

IL-2 shows multiple immunological effects and acts via ligation to the IL-2 receptor (IL-2R). The interactions between IL-2Rα (CD25), IL-2Rβ (CD122), and IL-2Rγ (CD132) subunits result in the trimeric high-affinity IL-2Rαβγ. CD25 confers high-affinity binding to IL-2. In contrast, the β and γ subunits expressed on resting CD4^+^ and CD8^+^ T cells, NK cells, monocytes, and macrophages are involved in signal transduction (176). Bempegaldesleukin (NKTR-214), functioning as an IL-2 agonist targeting CD122, yielded remarkable results in combination with nivolumab. The combined therapy exhibited an exceptional ORR of 59.5% across diverse advanced solid tumors. Notably, this combination fostered heightened infiltration and activity of CD8^+^ T cells while not augmenting Tregs (177). IL-6, acknowledged for its versatile cytokine functions, emerged as a pivotal player in cancer-related processes (178).

IL-6-mediated inflammation could play a role in the adverse health outcomes associated with NSCLC, contributing to both morbidity and mortality. In preclinical and phase I and II clinical trials, ALD518, which targets IL-6, has shown promising results (179). Specifically, it is well-tolerated, which means that patients can handle the treatment without severe side effects. Furthermore, ALD518 appears to improve conditions related to NSCLC, such as anemia and cachexia. However, while ALD518 has shown positive effects on anemia and cachexia, other clinical outcomes still require further investigation (179). These potential outcomes include its impact on OS, the tendency for blood to clot more efficiently, which is associated with lung cancer, and whether it affects the resistance of the tumor to inhibitors targeting the EGF pathway.

Moreover, patients with lower baseline IL-6 levels responded more favorably to PD-1/PD-L1 inhibitors in NSCLC, showcasing elevated ORR and prolonged OS and PFS. The strategic targeting of IL-6, particularly with anti-CTLA-4 therapy, demonstrated promise in enhancing survival outcomes (180–182). Consequently, combining IL-6 inhibitors with ICIs may be a potential therapeutic approach for patients with NSCLC.

Pegilodecakin, a PEGylated IL-10 variant, can induce CD8^+^ T cells and stimulate immune responses (183). When combined with anti-PD-1 therapy, pegilodecakin increased the expansion of specific CD8^+^ T-cell subsets. The efficacy of combining pegilodecakin (AM0010) with anti-PD-1 treatment was evaluated in previously treated NSCLC patients (184). This study emphasized the factors influencing responses to PD-1/PD-L1 axis-targeting agents in NSCLC, including PD-L1 expression, TMB, interferon-associated mRNA expression profile (GEP), and the absence of liver metastases. Pegilodecakin, recognized for its ability to stimulate the survival and expansion of intratumoral CD8^+^ T cells, was proposed as a potential augmentation to anti-PD-1 therapy. The methods involved treating NSCLC subjects with pegilodecakin alongside either pembrolizumab or nivolumab, and subsequent response assessments revealed a 41% ORR with 11 partial responses (PRs) and 46% stable disease (SD). Significantly, positive responses were observed in scenarios where anti-PD-1 therapy traditionally exhibited limited efficacy, including instances of low PD-L1 expression, low TMB, and the presence of liver metastasis. The study concluded that incorporating pegilodecakin into anti-PD-1 therapy demonstrated promising response rates and sustained benefits, advocating further exploration in larger-scale studies. This suggests a potential role for pegilodecakin in enhancing the effectiveness of anti-PD-1 treatments for advanced NSCLC patients (184).

In a phase 1b trial, the authors explored the safety, tolerability, and efficacy of combining the anti-PD-1 monoclonal antibody nivolumab with the IL-15 superagonist ALT-803 in patients with previously treated NSCLC (185). Given the limited success of PD-1/PD-L1 blockade in NSCLC, the study aimed to assess the potential of agonists targeting the shared IL-2 and IL-15Rβγ pathway. Twenty-three patients were enrolled, with 21 receiving treatments across four ALT-803 doses. No dose-limiting toxicities were observed, and the MTD was not reached. Frequent AE included injection-site reactions and flu-like symptoms, with grade 3 events like lymphocytopenia and fatigue occurring in two patients each. Notably, a grade 3 myocardial infarction occurred in one patient, but no grade 4 or 5 AEs were reported. The recommended phase 2 dose was determined to be 20 μg/kg ALT-803 subcutaneously once per week, paired with 240 mg intravenous nivolumab every 2 weeks. These results indicate that this combination exhibited promising clinical activity and safety, suggesting potential antitumor efficacy for this novel class of agents in relapsed and refractory NSCLC (185). These findings indicate that monotherapy with IL‐15 can induce an inadequate immune response against the tumor cells. Therefore, combining IL‐15 with IL‐15Rα or ICIs can augment the antitumor immune response.

A phase II pilot trial from September 2003 to November 2006 explored the potential synergistic effects of combining IL-2 with gefitinib for treating advanced, progressive NSCLC. The study involved 70 consecutive patients who had previously undergone chemotherapy. The patients were divided into two groups: the first 39 received gefitinib alone (G group), while the remaining 31 received both gefitinib and subcutaneous IL-2 (GIL-2 group). IL-2 was administered twice a day on days 1 and 2 and once daily on days 3, 4, and 5 every week for four consecutive weeks with a 4-week rest period. The results indicated that the combination therapy showed promising outcomes. The GIL-2 group exhibited a higher overall response rate, disease control rate, and median OS than the G group. Grade 3–4 toxicities were observed, with skin rash predominant in the gefitinib group and fever in the IL-2 group. Notably, skin toxicity and the use of IL-2 were independently associated with an improvement in survival. These findings suggest that adding IL-2 enhanced the efficacy of gefitinib in the treatment of advanced NSCLC, underscoring its potential as a therapeutic strategy (186).

Combining ILs with their modulators with ICIs offers a promising frontier in the treatment landscape for advanced solid tumors. These findings stimulate further research avenues and hold the potential for developing innovative therapeutic strategies in cancer treatment. **Table 3** exhibits the data presented in this section.

**Table 3:** The most important combination therapies using cytokines for treating patients with NSCLC. .

Intervention	Type of NSCLC and study	Outcome	Ref/NCT
**Bempegaldesleukin (NKTR-214) + nivolumab**	A single-arm, phase I dose-escalation trial	○ **Dose-limiting toxicities:** ○ Three dose-limiting toxicities were reported during dose escalation in 2 of 17 patients. ○ Hypotension (n = 1) ○ Hyperglycemia (n = 1) ○ Metabolic acidosis (n = 1) ○ **Common treatment-related adverse events** (TRAEs): ○ The most common TRAEs included: ○ Flu-like symptoms (86.8%) ○ Rash (78.9%) ○ Fatigue (73.7%) ○ Pruritus (52.6%) ○ **Grade 3/4 TRAEs:** ○ Eight patients (21.1%) experienced grade 3/4 TRAEs. ○ **Treatment-related deaths:** ○ There were no treatment-related deaths. ○ **ORR:** ○ The total ORR across tumor types and dose cohorts was 59.5% (22/37). ○ Complete response (CR): 7 (18.9%). ○ **Immune response analysis:** ○ Cellular and gene expression analysis of longitudinal tumor biopsies revealed increased infiltration, activation, and cytotoxicity of CD8^+^ T cells. ○ No enhancement of regulatory T cells was observed. ○ **Recommended phase II dose:** ○ The combination was well tolerated at the recommended phase II dose (BEMPEG 0.006 mg/kg plus nivolumab 360 mg every 3 weeks). ○ **Clinical activity:** ○ The combination demonstrated encouraging clinical activity irrespective of baseline PD-L1 status.	(177) NCT02983045
**Pegilodecakin + pembrolizumab or nivolumab**	NSCLC patients	○ **ORR:** ○ Pegilodecakin + pembrolizumab group: 41% (11 partial responses) ○ Disease control rate (DCR): 87% (11 PRs + 12 stable disease) ○ **PD-L1 expression:** ○ PD-L1 < 1% (PD-L1-negative): ▪ 4 of 4 PRs in pegilodecakin + pembrolizumab group ▪ 4 of 8 PRs in pegilodecakin + nivolumab group ○ **TMB and GEP:** ○ 5 of 8 subjects with low to intermediate TMB (<243 mut) had a PR. ○ 2 of 6 GEP-negative subjects had a PR. ○ **Metastasis and response:** ○ 5 of 8 subjects with liver metastasis had a PR. ○ **PFS and OS:** ○ Pegilodecakin + pembrolizumab group: ▪ mPFS: 10.9 months ▪ mOS: 32.2 months ○ Pegilodecakin + nivolumab group: ▪ mPFS: not reached ▪ mOS: not reported (not reached)	(184)
**Nivolumab + IL-15 superagonist ALT-803**	Patients with previously treated NSCLC phase 1b trial	○ **Dose-limiting toxicities:** none recorded ○ **Maximum tolerated dose:** not reached ○ **Adverse events:** ○ **Most common:** injection-site reactions (90% of 21 patients) ○ **Second most common:** flu-like symptoms (71% of 21 patients) ○ **Grade 3 adverse events:** lymphocytopenia and fatigue (each occurred in two patients), myocardial infarction (occurred in one patient) ○ **Grade 4 or 5 adverse events:** none recorded	(185) NCT02523469
**IL-2 + gefitinib**	Patients with previously treated NSCLC A phase II pilot trial	○ **Gefitinib toxicity (grade 3–4):** ○ Skin rash: 7% ○ Asthenia/anorexia: 6% ○ Diarrhea: 7% ○ **IL-2 treatment toxicity (grade 2–3):** ○ Fever: 46% ○ Fatigue: 21% ○ Arthralgia: 13% ○ **GIL-2 group:** ○ Overall response rate: 16.1% (6.4% complete response) ○ Disease control rate: 41.9% ○ Median time to progression: 3.5 months (95% CI, 3.2–3.8) ○ Median OS: 20.1 months (95% CI, 5.1–35.1) ○ Actuarial 1-year survival rate: 54% ○ **G group:** ○ Overall response rate: 5.1% (only partial response) ○ Disease control rate: 41% ○ Median time to progression: 4.1 months (95% CI, 2.6–5.7) ○ Median OS: 6.9 months (95% CI, 4.9–8.9) ○ Actuarial 1-year survival rate: 30% ○ **Independent associations with survival improvement:** ○ Skin toxicity (p < 0.001; HR = 0.29; 95% CI, 0.16–0.54) ○ Use of IL-2 (p < 0.001; HR = 0.33; 95% CI, 0.18–0.60)	(186)

NSCLC, non-small cell lung cancer; ORR, objective response rate; PD-L1, programmed death-ligand 1; PR, partial response; TMB, tumor mutational burden; mPFS, median progression-free survival; mOS, median overall survival.

## 6 Challenges and considerations

Combination therapies for NSCLC represent a cutting-edge approach in oncology, leveraging the synergistic effects of multiple drugs to enhance treatment efficacy. However, this innovative strategy has challenges, side effects, and crucial considerations that require thorough examination (187).

One of the primary challenges in developing and implementing combination therapies for NSCLC lies in managing the heightened risk of toxicity (188). When multiple drugs are administered simultaneously, the potential for AEs increases, posing a delicate balance between achieving therapeutic goals and maintaining patient wellbeing (188). The challenge is compounded by NSCLC patients often presenting with diverse clinical profiles, necessitating personalized treatment plans considering individual tolerances and susceptibilities to AEs (108). The toxicity associated with combination therapies can manifest in various ways, including hematologic, gastrointestinal, and dermatologic side effects (189). Hematologic toxicity, such as myelosuppression leading to anemia, neutropenia, or thrombocytopenia, can compromise the patient’s immune system and overall wellbeing (190). Gastrointestinal side effects, including nausea, vomiting, and diarrhea, are common concerns that can significantly impact a patient’s quality of life (106). Dermatologic toxicities, such as rash or skin irritation, further underscore the need for meticulous monitoring and intervention (191). In a single-center retrospective cohort study involving 354 adult patients with NSCLC receiving ICI therapy between 2014 and 2018, the impact of irAEs, the timing of these events, and prior targeted TKI therapy on clinical outcomes were investigated (192). The study aimed to assess OS and real-world PFS outcomes and develop predictive models using various approaches. The results revealed that patients experiencing irAEs had significantly longer OS and real-world PFS (rwPFS) compared to those who did not experience these events. Specifically, the median OS was 25.1 months vs. 11.1 months, and the median rwPFS was 5.7 months vs. 2.3 months for patients with and without irAEs, respectively.

Additionally, patients who received TKI therapy before initiating ICI demonstrated significantly shorter OS than patients without prior TKI therapy (median OS 7.6 months vs. 18.5 months). After adjusting for other variables, irAEs and initial TKI therapy were significant predictors of both OS and rwPFS. The study also highlighted the comparable performance of logistic regression and machine learning approaches in predicting 1-year OS and 6-month rwPFS. Therefore, the occurrence of irAEs, the timing of these events, and prior TKI therapy were significant predictors of survival in NSCLC patients treated with ICIs. These findings suggest the need for future prospective studies to investigate further the impact of irAEs and the therapy sequence on the survival outcomes of NSCLC patients undergoing ICI treatment (192).

A study discusses the tolerability and unique toxicities associated with ICIs, focusing on irAEs (193). While ICIs are generally well-tolerated, they can lead to dysregulation in normal immune self-tolerance, resulting in inflammatory side effects. The impact of irAEs is widespread, affecting various organ systems, with the gastrointestinal tract, endocrine glands, skin, and liver being the most commonly involved. Notably, these AEs can manifest at any point during treatment and, in rare cases, even after completion. Research by Owen and colleagues indicates that approximately 30% of patients with NSCLC treated with ICIs develop irAEs (194). The Kichenadasse et al. comprehensive evaluation of multiorgan irAEs underscores the need for more extensive information. Managing irAEs requires careful differentiation between infectious causes and symptom progression, and close collaboration with disease-specific subspecialties is recommended. Corticosteroids emerge as the primary treatment for most irAEs, emphasizing the importance of early intervention in the general management of immune-mediated toxicity. Grade 1–2 irAEs can be monitored closely, while certain endocrine irAEs may be treated with hormone supplementation without resorting to corticosteroids. For severe cases (grade 3–4), moderate-to-high-dose steroids and additional immunosuppressants like tocilizumab and cyclophosphamide may be necessary. The findings also highlight a growing area of interest: irAEs after immunotherapy rechallenge. Dolladille and colleagues found that rechallenging with the same ICIs was associated with approximately 25%–30% of the same irAEs experienced previously. However, caution is warranted in interpreting these findings, and further pooled analyses are suggested before drawing definitive conclusions about the safety of ICIs in rechallenge scenarios (193).

Additionally, identifying the optimal combination of drugs poses a formidable challenge (195). The genetic and molecular heterogeneity of NSCLC makes it challenging to predict how individual patients will respond to specific therapeutic agents. Biomarker-driven approaches, which involve analyzing genetic mutations or expression patterns in a patient’s tumor, offer a promising avenue for tailoring combination therapies to the unique characteristics of each case (196). However, the complexity of these molecular profiles and the evolving nature of cancer cells require ongoing research and technological advancements to refine and expand our understanding of effective drug combinations. Moreover, the economic implications of NSCLC combination therapies must be considered. The cost of developing, producing, and administering multiple drugs concurrently contributes to the overall financial burden of cancer care (197). Access to these therapies may be limited, particularly in regions with constrained healthcare resources, raising essential considerations regarding healthcare equity. Addressing these economic challenges requires a concerted effort from stakeholders, including pharmaceutical companies, healthcare providers, and policymakers, to balance innovation and affordability (198).

Precision medicine, guided by comprehensive genomic profiling and the identification of predictive biomarkers, emerges as a critical component in navigating the challenges associated with NSCLC combination therapies (199, 200). By tailoring treatment strategies to the unique genetic makeup of each patient’s tumor, precision medicine holds the promise of optimizing therapeutic outcomes while minimizing the risk of adverse effects (201). Ongoing research endeavors, such as large-scale genomic profiling initiatives and clinical trials focused on biomarker-driven therapies, contribute valuable insights that fuel the evolution of precision medicine in NSCLC treatment.

As researchers and clinicians delve deeper into the intricacies of NSCLC combination therapies, the importance of patient-reported outcomes (PROs) becomes increasingly evident. PROs provide a direct and valuable source of information regarding the patient’s experience, capturing aspects of treatment impact that may not be apparent through traditional clinical assessments (202). Integrating PROs into clinical trials and routine practice allows for a more comprehensive understanding of the benefits and challenges associated with combination therapies, ultimately shaping treatment approaches that prioritize both efficacy and patient wellbeing.

The role of the TME in NSCLC combination therapies is another multifaceted consideration. The TME comprises various cell types, signaling molecules, and extracellular matrix components interacting with cancer cells (203). The dynamic interplay within the TME influences tumor progression, response to treatment, and the development of resistance mechanisms. Understanding and manipulating the TME to enhance the efficacy of combination therapies is a burgeoning area of research (204). Strategies aimed at modulating the immune response, targeting angiogenesis, and disrupting supportive stromal elements within the TME hold promise in optimizing the outcomes of NSCLC combination therapies (204). Clinical trial design is pivotal in advancing our understanding of NSCLC combination therapies. Well-designed trials consider the complexities of the disease, incorporate biomarker-driven approaches, and account for the dynamic nature of cancer cells (28, 205). Adaptive trial designs, which allow for modifications based on interim data analyses, offer flexibility in optimizing treatment regimens (206). Collaboration between researchers, clinicians, and pharmaceutical companies is essential to design trials that meet regulatory standards and address the practical challenges encountered in real-world clinical settings (108).

An investigation emphasized the necessity for further research into the molecular aspects of NSCLC pathobiology (207). The objective was to identify critical molecular targets that influence tumor immunity, enabling the development of strategic therapeutic combinations to enhance the effectiveness of ICIs. Particular attention was given to Yes-associated Protein (YAP) and transcriptional coactivator with PDZ-binding motif (TAZ), identified as the ultimate effectors of the Hippo signaling transduction pathway. The study suggested that these proteins play a pivotal role in NSCLC development and progression, specifically in the context of immune evasion. The proposed therapeutic approach involves investigating the potential synergistic effects of YAP/TAZ inhibitory agents and ICIs for managing NSCLC patients. This strategy aimed to overcome resistance issues associated with current immunotherapies by targeting molecular pathways linked to NSCLC development. By inhibiting YAP/TAZ, researchers sought to modulate the immune response against NSCLC tumors, potentially amplifying the efficacy of ICIs (207). These findings indicate the persistent challenges in treating NSCLC despite advancements in ICIs. It emphasizes the importance of comprehending the molecular intricacies of NSCLC, mainly focusing on the role of YAP and TAZ in immune evasion. The proposed strategy of combining YAP/TAZ inhibitors with ICIs holds promise as a potential avenue for developing more potent therapeutic approaches in NSCLC management.

A study discussed the prevalence of *MET* amplification causing resistance to EGFR TKIs in *EGFR*-mutated NSCLC patients (208). To tackle this resistance, researchers have explored combined treatment with *EGFR* TKIs and *MET* TKIs, but the emergence of acquired resistance limits durable responses. In response to these challenges, a novel antibody–drug conjugate called REGN5093-M114, targeting MET in MET-driven patient-derived models, has been investigated for its preclinical activity. Using patient-derived organoids, patient-derived cells, and American Type Culture Collection (ATCC) cell lines, the study reveals that REGN5093-M114 exhibits significant antitumor efficacy compared to MET TKIs or unconjugated METxMET biparatopic antibody (REGN5093). Significantly, REGN5093-M114’s effectiveness is not restricted by the MET gene copy number, indicating its potential to address resistance in a broad range of cases. The study emphasized the positive response of MET-overexpressed TKI-naïve *EGFR*-mutant NSCLC cells to REGN5093-M114 treatment, suggesting its capability to complement EGFR TKIs in scenarios where they may fall short. The outcomes of this study indicated the predictive power of cell surface MET expression in determining the efficacy of REGN5093-M114.

Furthermore, REGN5093-M114 demonstrated potency in reducing tumor growth in *EGFR*-mutant NSCLC cases with specific genetic alterations, such as PTEN loss or *MET Y1230C* mutation, especially after previous treatment with osimertinib and savolitinib. This highlights REGN5093-M114’s potential as a promising approach to overcoming acquired resistance challenges in these cases. REGN5093-M114, as a promising candidate, may address resistance mechanisms linked to MET amplification in *EGFR*-mutated NSCLC. The antibody–drug conjugate proves effective across various genetic backgrounds, showcasing its adaptability in overcoming resistance. These findings suggest that REGN5093-M114 could be a valuable addition to the treatment options for patients encountering challenges associated with functional MET pathway blockade (208).

## 7 Concluding remarks

In conclusion, the landscape of NSCLC combination therapies is marked by remarkable potential and complex challenges. Managing the heightened risk of toxicity, identifying optimal drug combinations, addressing economic considerations, and navigating the psychosocial impact on patients are integral aspects of this evolving field. Precision medicine, immunotherapy, and an understanding of the TME represent critical pillars in shaping the future of NSCLC combination therapies. As research continues to unravel the intricacies of this disease, a multidisciplinary and patient-centered approach will be crucial in realizing the full therapeutic potential of combination strategies for NSCLC.

## Author contributions

YW: Writing – original draft. GY: Writing – original draft, Writing – review & editing. KJ: Writing – original draft, Writing – review & editing. JQ: Writing – original draft, Writing – review & editing.
